# *Mycobacterium abscessus* virulence traits unraveled by transcriptomic profiling in amoeba and macrophages

**DOI:** 10.1371/journal.ppat.1008069

**Published:** 2019-11-08

**Authors:** Violaine Dubois, Alexandre Pawlik, Anouchka Bories, Vincent Le Moigne, Odile Sismeiro, Rachel Legendre, Hugo Varet, María del Pilar Rodríguez-Ordóñez, Jean-Louis Gaillard, Jean-Yves Coppée, Roland Brosch, Jean-Louis Herrmann, Fabienne Girard-Misguich

**Affiliations:** 1 Université Paris-Saclay, UVSQ, Inserm, Infection et inflammation, Montigny-Le-Bretonneux, France; 2 Institut Pasteur, Unité de Pathogénomique Mycobactérienne intégrée, UMR3525 CNRS, Paris, France; 3 Institut Pasteur—Bioinformatics and Biostatistics Hub—C3BI, USR 3756 IP CNRS, Paris, France; 4 Institut Pasteur—Transcriptome and Epigenome Platform—Biomics Pole—CITECH, Paris, France; 5 Laboratoire d'Écologie, Systématique et Évolution, Université Paris-Saclay, Orsay, France; 6 AP-HP. GHU Paris Saclay, Hôpital Ambroise Paré, Boulogne Billancourt, France; 7 AP-HP. GHU Paris Saclay, Hôpital Raymond Poincaré, Garches, France; McGill UniversityHealth Centre, CANADA

## Abstract

Free-living amoebae are thought to represent an environmental niche in which amoeba-resistant bacteria may evolve towards pathogenicity. To get more insights into factors playing a role for adaptation to intracellular life, we characterized the transcriptomic activities of the emerging pathogen *Mycobacterium abscessus* in amoeba and murine macrophages (Mϕ) and compared them with the intra-amoebal transcriptome of the closely related, but less pathogenic *Mycobacterium chelonae*. Data on up-regulated genes in amoeba point to proteins that allow *M*. *abscessus* to resist environmental stress and induce defense mechanisms, as well as showing a switch from carbohydrate carbon sources to fatty acid metabolism. For eleven of the most upregulated genes in amoeba and/or Mϕ, we generated individual gene knock-out *M*. *abscessus* mutant strains, from which ten were found to be attenuated in amoeba and/or Mϕ in subsequence virulence analyses. Moreover, transfer of two of these genes into the genome of *M*. *chelonae* increased the intra-Mϕ survival of the recombinant strain. One knock-out mutant that had the gene encoding Eis N-acetyl transferase protein (*MAB_4532c*) deleted, was particularly strongly attenuated in Mϕ. Taken together, *M*. *abscessus* intra-amoeba and intra-Mϕ transcriptomes revealed the capacity of *M*. *abscessus* to adapt to an intracellular lifestyle, with amoeba largely contributing to the enhancement of *M*. *abscessus* intra-Mϕ survival.

## Author summary

The emerging pathogen *Mycobacterium abscessus* differs from other rapidly growing mycobacteria by its ability to survive phagocytosis by human macrophages. These virulence traits render *M*. *abscessus* pathogenic in vulnerable hosts with structural lung disease. How patients become infected with *M*. *abscessus* remains unclear, as *M*. *abscessus* is not or only very rarely isolated from the environment. Co-culturing of *M*. *abscessus* and amoeba increases the virulence of the bacteria for subsequent mouse infection, but little is known about *M*. *abscessus* virulence in general. Until now, the prediction of *M*. *abscessus* virulence factors was essentially based on the comparative analysis of the genome of *M*. *abscessus* with that of *Mycobacterium chelonae*, a closely related mycobacterium that occasionally causes opportunistic human infections, but to a much lesser extent than *M*. *abscessus*. Here, by transcriptomic and functional approaches, we decipher a global view on *M*. *abscessus* adaptation to intracellular life.

## Introduction

To date, most of the known mycobacterial species are environmental organisms found in soil [1], air [2] and water [3–5], and belong to the Rapid Growing Mycobacteria (RGM). In contrast, pathogenic mycobacteria mostly belong to the Slow Growing Mycobacteria (SGM), although some exceptions exist, as for example *Mycobacterium abscessus*, an emerging mycobacterial pathogen that is causing serious infections in patients with cystic fibrosis or other structural lung diseases. *M*. *abscessus* is member of the *Mycobacterium chelonae* complex, which includes *M*. *abscessus*, *M*. *chelonae* and *Mycobacterium immunogenum*. Together with *Mycobacterium fortuitum*, the *M*. *chelonae* complex members represent the main opportunistic pathogens among RGM [6–8].

Compared to other Non-Tuberculous Mycobacteria (NTM), recovery of *M*. *abscessus* from the environment is rare [9]. However, information from its genome sequence suggests the presence of the bacterium at the interface of soil, vegetation and water, an environment where free-living amoebae (FLA) are commonly found [10]. FLA have been isolated from habitats in common with mycobacteria [11,12] including cold-drinking water systems [13,14], hot water systems in hospitals and cooling towers [15]. FLA are ubiquitous organisms that feed on bacteria, and these bacteria have likely developed adaptations to the intracellular lifestyle to become Amoeba-Resistant Bacteria (ARB) [16,17]. Mycobacteria have been isolated from such habitats by amoebal enrichment techniques [18,19], suggesting that horizontal gene transfer and adaptation to an intracellular lifestyle might also take place in such an environment [20–22]. Finally, amoebae are often considered as an ancestral form of macrophages (Mϕ) sharing similar cellular structures and biological features [23–25].

*M*. *abscessus* has been shown to be resistant to amoeba phagocytosis and encystment, a property shared with all mycobacteria with the exception of the attenuated *M*. *bovis* BCG vaccine strain [16,26,27]. In addition, co-culture of *M*. *abscessus* with *Acanthamoeba castellanii* (Ac) increases its virulence when subsequently used for aerosol infection in the mouse model [27]. Similarly, co-culture of amoebae with *M*. *avium* was found to trigger *M*. *avium* virulence by enhancing both entry and intracellular multiplication of the bacterium [28]. The essential role of the ESX-4 *M*. *abscessus* type VII secretion system (T7SS) has also been demonstrated based on an intra-amoebal viability screen of *M*. *abscessus*, unraveling the active role of ESX-4 in intracellular survival of *M*. *abscessus* [29]. Taken together, it seems likely from reports in the literature that *M*. *abscessus* has contact to FLA as part of its natural lifecycle, which however is only poorly explored to date.

In order to gain deeper insights into this individual lifestyle of *M*. *abscessus*, we sought for ways to characterize the virulence traits and decided to study the transcriptional signatures of *M*. *abscessus* in Ac and Mϕ, in comparison to the ones of *M*. *chelonae*, with the aim of identifying potential adaptations of *M*. *abscessus* to intracellular life and to pathogenicity. Although amoebae and Mϕ share common features, it has been shown that an intra-amoebal life requires specific adaptations [29]. A full description and analysis of *M*. *abscessus* transcriptomes shall allow a complete picture of *M*. *abscessus* intracellular replication and survival mechanisms to be obtained, both in an amoeba environmental host and in Mϕ.

## Results

### Overall description of the *M. abscessus* intracellular transcriptomes

**Fig 1:**
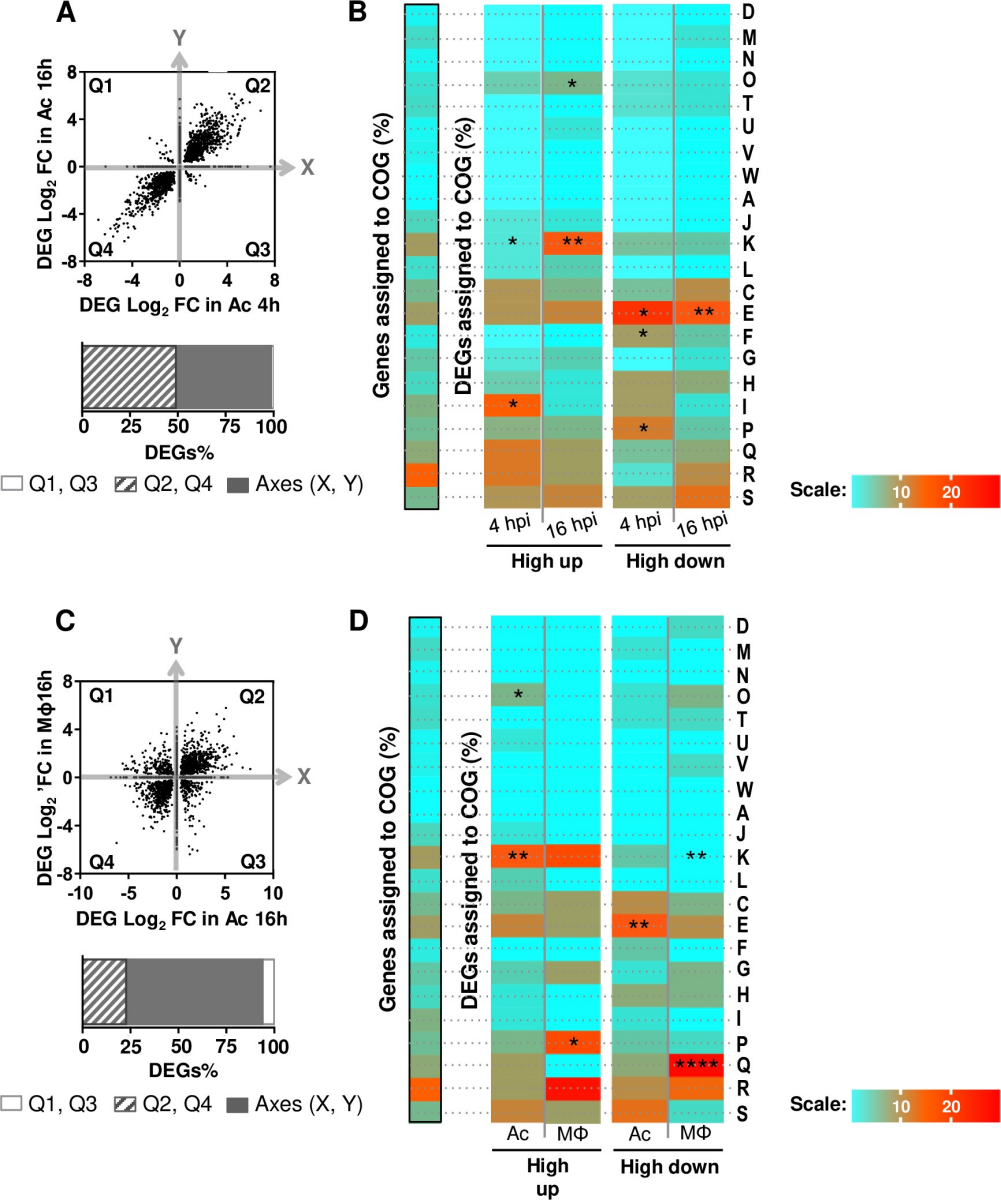
**Description of *M*. *abscessus* transcriptomes in Ac (A, B) and Mϕ (C, D).** Differentially expressed genes (DEGs) from comparisons of co-cultures of mycobacteria with *A*. *castellanii* (Ac) and macrophages (Mϕ) relative to planktonic growth were categorized according to their fold change (FC) expressed in Log_2._ DEGs Log_2_ fold change (FC) in Ac 4 hpi (X axis) are plotted against DEGs FC 16 hpi (Y axis) (A) DEGs Log_2_ FC in Ac 16 hpi (X axis) are also plotted against DEGs FC in macrophages (Mϕ) 16 hpi (Y axis) (C). Each dot on the graph corresponds to a DEG. DEGs from quadrant 2 (Q2) and 4 (Q4) are genes regulated in the same direction whereas DEGs from Q1 and Q3 are genes regulated in opposite direction. Dots on the plot axes (X, Y) are genes regulated in one condition only. Proportions of DEGs of Q1, Q2, Q3, Q4 and axis (X, Y) are quantified in the histogram. *M*. *abscessus* adaptations to Ac (B) and Mϕ (D) unraveled by COG categorization. Highly regulated genes (Log_2_FC > |4|) were assigned to COGs. The genome assignation (framed in black) serves as a reference for COG enrichment tests. Fisher’s exact tests were performed to compare the transcriptome sets and the genome set of gene assignments to COG. Assignments are expressed in percentages and depicted with a scale from light blue (lower percentages) to red (higher percentages). If the DEGs percentage for a COG category is significantly lower than that of the genome, the COG is under-represented. On the contrary, if the DEGs percentage for a COG category is significantly higher than that of the genome, the COG is over-represented. D: Cell cycle control, cell division, chromosome partitioning, M: Cell wall/membrane/envelope biogenesis, N: Cell motility, O: Post-translational modification, protein turnover, chaperones, T: Signal transduction mechanisms, U: Intracellular trafficking, secretion, and vesicular transport, V: Defense mechanisms, W: Extracellular structures, A: RNA processing and modification, J: Translation, ribosomal structure and biogenesis, K: Transcription, L: Replication, recombination and repair, C: Energy production and conversion, E: Amino acid transport and metabolism, F: Nucleotide transport and metabolism, G: Carbohydrate transport and metabolism, H: Coenzyme transport and metabolism, I: Lipid transport and metabolism, P: Inorganic ion transport and metabolism, Q: Secondary metabolites biosynthesis, transport and catabolism, R: General function prediction only, S: Function unknown. * *p*<0.05, ** *p*<0.01, *** *p*<0.001, **** *p*<0.0001.

RNAseq data from *M*. *abscessus* planktonic or intracellular cultures (3 to 4 replicates per condition) were analyzed and compared to identify *M*. *abscessus* genes that were up- or down-regulated after Ac and Mϕ co-cultures. Differentially expressed genes (DEG) are thus defined as genes for which expression changes between intracellular and planktonic growth. Transcriptomes of *M*. *abscessus* in Ac 4 and 16 hours post-infection (hpi), in Mϕ 16 hpi and transcriptomes of *M*. *chelonae* in Ac 16 hpi were obtained and invariant genes were excluded from the analyses. Normalization and hierarchical clustering of normalized raw data confirmed the technical quality of transcriptomes (**S1 Fig**). DEGs were identified using the *DESeq2* package [30] (**S1 Table**) and the fold change (FC) values were recorded. In particular, Log_2_ fold change (Log_2_FC) values from *M*. *abscessus* transcriptomes were then compared. In Ac, most DEGs up-regulated or down-regulated at 4 hpi were still up- or down-regulated at 16 hpi (**Fig 1A**). In order to detail the biological changes that correlated with *M*. *abscessus* intracellular regulation, we first grouped the DEGs into clusters of orthologues (Cluster of Orthologous Groups or COGs) [31]. Highly up-regulated genes (Log_2_FC > 4) in Ac were more frequently found in COG O (Post-translational modification, protein turnover, chaperones), COG K (Transcription) and COG I (Lipid transport and metabolism) compared to the genome reference, although this last category tended to be under-represented at 16 hpi, suggesting that the percentage of DEGs allocation for this category is lower than that from the genome allocation (**Fig 1B**). Comparatively, highly down-regulated genes (Log_2_FC < -4) were assigned to COG E and COG F (amino-acid and nucleotide transport and metabolism respectively) (**Fig 1B**).

At first glance, exposure of *M*. *abscessus* to the intra-amoebal and intra-macrophagic environments generated similar gene regulation patterns. Indeed, by comparing *M*. *abscessus* Ac-16hpi *vs*. Mϕ-16hpi transcriptomes, we show that only 20% of DEGs were regulated in the opposite direction (increased versus decreased and vice versa) and only 10% were specific to Ac or Mϕ (**Fig 1C**). The representation of DEGs according to their FC patterns highlighted that most DEGs showed low changes in both Ac and Mϕ (Log_2_FC < |2|) 16 hpi (**S2A Fig**). Nonetheless, in comparison to the situation in Mϕ, large variations of gene expression patterns were observed in Ac, the percentage of genes with a FC over 4 (Log_2_FC > |2|) being 4 times higher than in Mϕ (**S2A Fig**). In addition, up-regulated DEGs predominated in Ac in comparison to the in-Mϕ environment (**S2B Fig**). Thus, the intra-amoebal environment seems more prone to large variations and in particular to strong induction of genes compared to the in-Mϕ environment.

COG assignments highlighted the differences between *M*. *abscessus* high DEGs in Mϕ and Ac (**Fig 1D**). COG O, which was over-represented in the *M*. *abscessus* highly up-regulated genes in Ac, was more frequently associated with highly down-regulated genes in Mϕ (**Fig 1B and 1D**). By comparison, COG P (Inorganic ion transport and metabolism) was over-represented in the *M*. *abscessus* highly up-regulated genes in Mϕ only, potentially illustrating different adaptations to amoebal and Mϕ environments (**Fig 1D**).

### Main biological pathway changes of *M. abscessus* in Ac and Mϕ

**Fig 2:**
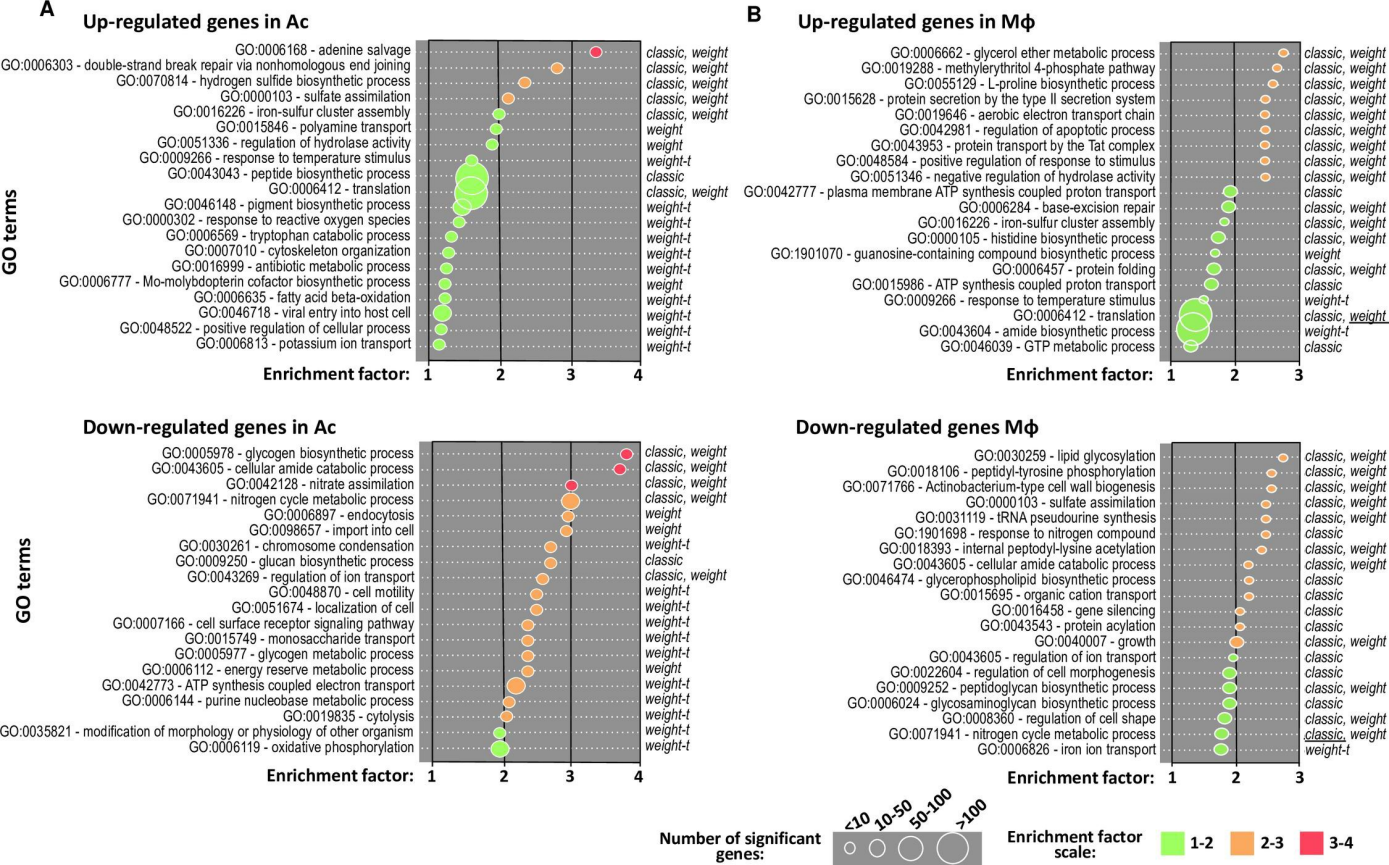
Gene ontology enrichment analyses applied on *M*. *abscessus* intracellular transcriptomes. **Differentially expressed genes in Ac (A) and in M**ϕ **(B) 16 hpi.** GO enrichment analysis was performed with the topGO R package [72]. Enriched GOs are sorted according to their enrichment factor (EF), corresponding to the ratio of significant DEGs assigned to the GO over expected assigned DEGs to the GO as defined by topGO [72]. Enriched GOs are represented by circles which size is proportional to the amount of significant DEGs assigned. Positive statistical tests are given that face each GO. Method giving the smallest *p*-value is underlined.

We performed a gene ontology (GO) enrichment analysis to further characterize the *M*. *abscessus* adaptions in Ac (**Fig 2A**) and Mϕ (**Fig 2B**). GO enrichment data were qualified by an enrichment factor (EF) (1 to 4) and a number of significantly enriched genes (from small (<10) to large (>100)) (**Fig 2**).

In Ac, the most enriched up-regulated *M*. *abscessus* genes fell into polyamine transport (GO:0015846) to adenine salvage (GO:0006168), including small groups of genes involved in sulfur metabolism (sulfate assimilation pathway (SAP) (GO:0000103), hydrogen sulfide (H_2_S) biosynthetic pathway (GO:0070814) and detoxification (iron-sulfur cluster assembly (GO:0016226)) (**Fig 2A, upper panel**).

In Mϕ, *M*. *abscessus* up-regulated enriched genes fell into different GO in comparison to those up-regulated in Ac. L-proline biosynthetic process (GO:0055129); methylerythritol 4-phosphate MEP pathway (GO:0019288) and glycerol ether metabolic process (GO:0006662) were the most enriched (**Fig 2B, upper panel**). These GO are followed by the type II secretion system and notably the Tat (twin-arginine translocation) pathway.

In Ac *M*. *abscessus* infections, the most enriched down-regulated genes fell into the nitrate assimilation GO (GO:0042128), to glycogen biosynthetic process GO (GO:0005978) (**Fig 2A, lower panel**). In particular GO related to transport and metabolism of glucose were enriched (GO:0005977, GO:00015749, GO:0009250, GO:0005978) (**Fig 2A, lower panel**).

In Mϕ, *M*. *abscessus* down-regulated genes related to growth and parietal activities. From GO:0040007 corresponding to growth, up to GO:0030259 corresponding to lipid glycosylation, in addition to GO:0071941 (nitrogen cycle metabolism process), GO:0009259 (peptidoglycan synthesis) and GO:0022604 (regulation of cell morphogenesis), GO enrichment analysis indicated that *M*. *abscessus* slows down its energy-demanding metabolic processes and growth rate (**Fig 2B, lower panel**).

Taken together, these observations suggest that *M*. *abscessus* enters a slow-replicative state in Mϕ and dedicates its energy to detoxification and protein secretion into the host.

### Regulation of the central carbon metabolism of *M. abscessus* in Ac and Mϕ

**Fig 3:**
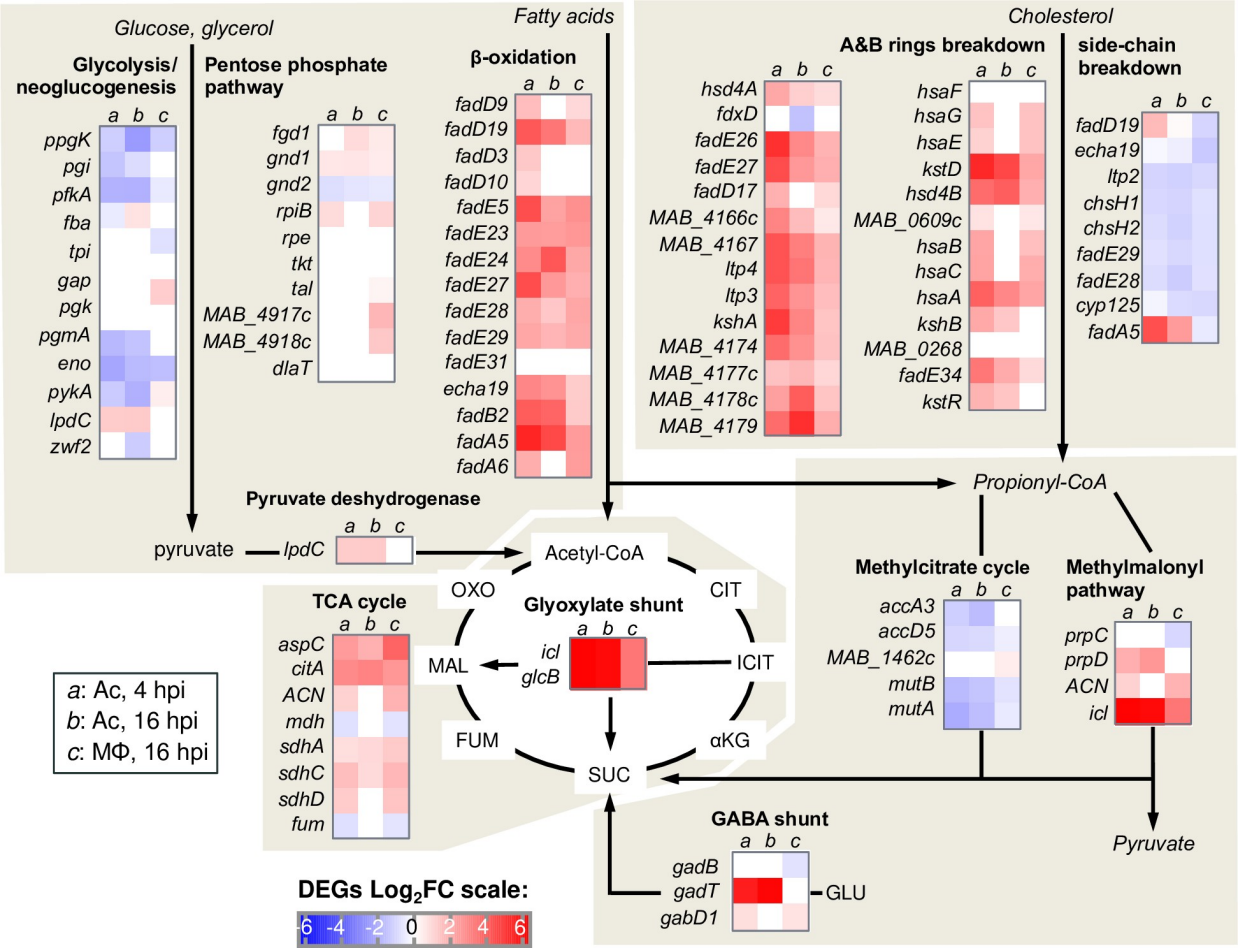
Intracellular *M*. *abscessus* relies on fatty acid and cholesterol catabolism. DEGs FC of genes implicated in Central Carbon Metabolism (CCM) is represented on a Heat Map ranging from blue (DOWN DEGs) to red (UP DEGs). On this Heat Map both *M*. *abscessus* intra-amoebal (Ac) and intra-macrophagic (Mϕ) DEGs are depicted.

Following the GO enrichment analysis, we investigated the different *M*. *abscessus* up- and down-regulated genes from metabolic pathways in Ac or Mϕ. The major finding was that *M*. *abscessus* switches from a simple sugar-based carbon source to fatty acids inside Ac and Mϕ (**Fig 3**). The glycolysis/neoglucogenesis and pentose phosphate pathways were mostly down-regulated or unchanged during intracellular growth, whereas the β-oxidation of fatty acids was up-regulated in Ac and Mϕ. This switch was observed from the early time points after Ac infection. Fifteen genes predicted to encode enzymes necessary for the biochemical activation and β-oxidation of fatty acids were up-regulated in Ac and Mϕ, such as: fatty acid-coenzyme A (CoA) synthase (*fadD3*, *9*, *10*, *19*); acyl-CoA dehydrogenase (*fadE5*, *14*, *23*–*24*, *27*–*29*, *31*); enoyl-CoA hydratase (*echA19*); hydroxy-butyryl-CoA dehydrogenase (*fadB2*) and acetyl-CoA transferase (*fadA5*, *6*). Genes implicated in the synthesis of enzymes involved in the breakdown of cholesterol A and B rings were highly induced in Ac and Mϕ. β-oxidation of fatty acid and cholesterol breakdown result in the accumulation of propionyl-CoA that is detoxified by the methylmalonyl pathway. By-products of these 3 pathways and the GABA shunt feed the TCA cycle. The succinate generated by the TCA cycle enables the bacterium to deal with anaerobic respiration [32]. In addition, *M*. *abscessus* may detoxify glyoxylate by converting it into malate via the glyoxylate shunt (**Fig 3**).

Furthermore, down-regulation of the mycolate operon (*MAB_2027*-*MAB_2039*) (**Table 1**), encompassing the β-ketoacyl-ACP synthases (KasA and KasB) and β-ketoacyl synthases (MAB_2031 & MAB_2029), as well as the malonyl-CoA acyl carrier protein transacylase (MCAT) homolog (MAB_2034), revealed that intracellular *M*. *abscessus* undergoes starvation as previously described [33].

**Table 1:** Regulation of *M*. *abscessus* mycolate synthesis operon in Ac and Mϕ.

Mma^a^ gene	Encoded protein	Mabs^b^ gene	FC Ac 4 hpi	FC Ac 16 hpi	FC Mϕ 16 hpi
*MYCMA_RS13950*	Acyl carrier protein	*MAB_2027*	-3.28 (3.15E-17)	-2.91 (3.08E-07)	NC^c^
*MYCMA_RS13945*	3-oxoacyl-ACP synthase (Kas B)	*MAB_2028*	-2.68 (3.42E-10)	-2.24 (8.40E-04)	NC
*MYCMA_RS13940*	Beta-ketoacyl synthase	*MAB_2029*	-2.95 (8.03E-11)	-4.48 (8.40E-04)	NC
*MYCMA_RS13935*	3-oxoacyl-ACP synthase (Kas A)	*MAB_2030*	-3.80 (8.03E-11)	-3.15 (3.94E-03)	-1.06 (5.38E-03)
*MYCMA_RS13930*	Beta-ketoacyl synthase	*MAB_2031*	-2.92 (1.02E-08)	NC	-1.85 (3.66E-03)
*MYCMA_RS13925*	3-oxoacyl-ACP reductase	*MAB_2032*	-2.16 (1.92E-05)	NC	NC
*MYCMA_RS13920*	Thioesterase	*MAB_2033*	-2.09 (3.06E-04)	NC	NC
*MYCMA_RS13915*	Malonyl CoA-ACP transacylase	*MAB_2034*	-2.09 (3.06E-04)	NC	NC
*MYCMA_RS13910*	Acyltransferase	*MAB_2035*	NC	NC	NC
*MYCMA_RS13905*	Membrane protein (MmpS)	*MAB_2036*	-1.16 (1.36E-02)	NC	-1.06 (6.55E-03)
*MYCMA_RS13900*	Hypothetical protein (MmpL)	*MAB_2037*	-0.68 (8.24E-03)	-0.86 (8.24E-03)	-0.72 (6.97E-07)
*MYCMA_RS13895*	Transporter	*MAB_2038*	NC	NC	NC
*MYCMA_RS13890*	Lipase (LipH)	*MAB_2039*	NC	0.86 (1.61E-02)	0.64 (7.73E-03)

^a^Mma gene: *Mycobacterium massiliense* gene (accession number NC_018150.2).

^b^Mabs gene: *Mycobacterium abscessus* gene (accession number NC_010397.1).

^c^NC: no change in gene expression

*P*-values are indicted in brackets.

### Regulation of putative virulence genes of *M. abscessus* in Ac and Mϕ

**Fig 4:**
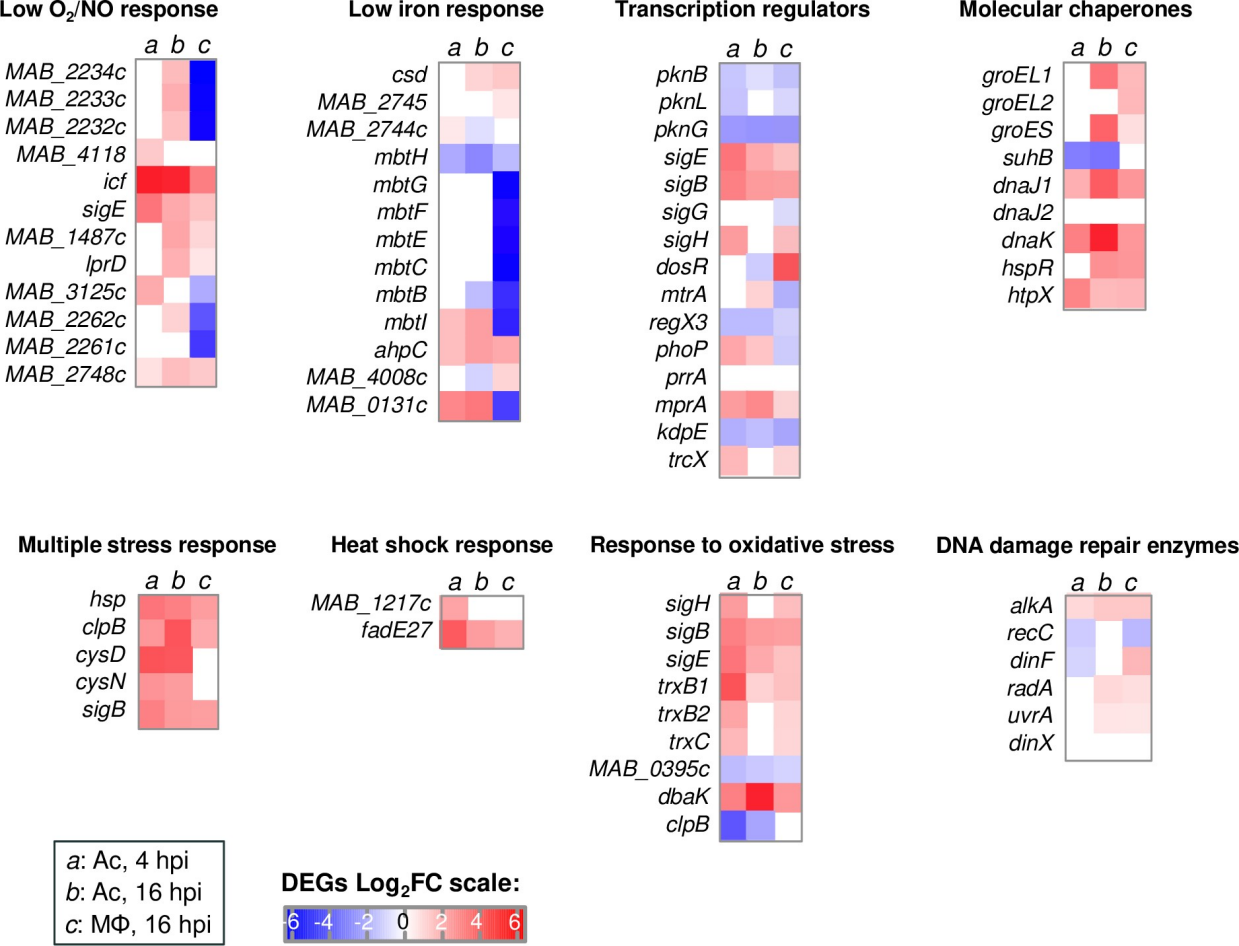
Regulation of genes required for pathogenic mycobacteria survival *in vitro*. Regulation of *M*. *abscessus* genes conserved in *M*. *tuberculosis*, known to be induced and to contribute to cellular microbicidal defenses of the tubercle bacillus in Mϕ [34] is represented on a Heat Map in a blue (repressed genes) to red (induced genes) color scale. On this Heat Map both *M*. *abscessus* intra-amoebal (Ac) and intra-macrophagic (Mϕ) DEGs are depicted and divided in categories: « broad transcription regulators » and genes implicated in the response to various intracellular stress (« Multiple stress response », « Heat Shock response », « Molecular chaperones », « DNA damage repair enzymes », « Response to low O_2_ / NO », « Low iron response », « Response to oxidative stress »).

We assessed the regulation of *M*. *abscessus* genes that show orthologs in *M*. *tuberculosis*, which were known to be induced and to contribute to the cellular microbicidal defenses of the tubercle bacillus in Mϕ [34]. The results obtained were similar for infection of Ac and Mϕ, with a few exceptions in the response to low O_2_/NO and in the low iron response (**Fig 4**). Transcriptional regulators such as *dosR*, *phoP* and *mtrA* were regulated in the opposite direction in Ac and Mϕ, with *phoP* and *mtrA* only induced in Ac, and *dosR* exclusively induced in Mϕ. Other genes, known to contribute to the survival of the bacterium in response to oxidative stress [35], comprising *ahpD*, *bcp*, *trxB1* and *2*, and *trxC* genes [36], in addition to *ahpC*, were up-regulated in Ac and Mϕ.

Altogether, these analyses suggest that the induced sets of genes in Ac reflect the main adaptation signatures for resistance to intracellular stress that were also shown to be induced in Mϕ.

### *M*. *abscessus* highly-induced genes in amoeba

Since we showed that the intra-amoebal environment is favorable to strong induction of a gene set that is thought to contribute to *M*. *abscessus* survival in phagocytic cells, the highest FC values of the *M*. *abscessus* transcriptome in Ac 4 and 16 hpi were chosen and then compared to the FC values obtained from the *M*. *abscessus* transcriptome in Mϕ and the *M*. *chelonae* transcriptome in Ac. This comparison highlighted 45 genes that were upregulated only in *M*. *abscessus* during infection of Ac (**S2 Table**), whereas we also found 38 genes that were most induced during infection of Mϕ (**S3 Table**). Based on these data sets, we selected the genes that were specifically induced in Ac, and constructed six different deletion mutants in the reference strain *M*. *abscessus* CIP 104536T (ΔOP1 to ΔOP6) (**Table 2 and S2 Table**). Moreover, based on the *M*. *abscessus* transcriptome data in Mϕ, we constructed additional five deletion mutants (ΔOP 7 to ΔOP11) targeting the most induced genes or genes implicated in the adaptation to intracellular stress (**Table 2 and S3 Table)**.

**Table 2:** Deleted operons in *M*. *abscessus* CIP 104536T.

Operon ID	Mabs^a^ gene	Protein encoded	IPS^b^ motif analysis on hypothetical proteins
OP1 (2231455)	*MAB_4664*	Hypothetical protein	No IPS
OP2 (395711)	*MAB_1242c*	Hypothetical protein	No IPS
	*MAB_1243c*	Hypothetical protein	IPS035568: ABC transporter. FecCD/TroCD-like
	*MAB_1244c*	Hypothetical protein	No IPS
	*MAB_1245c*	Hypothetical protein	No IPS
	*MAB_1246c*	Hypothetical protein	No IPS
	*MAB_1247c*	Hypothetical protein	IPS005531: Alkaline shock protein Asp23
OP3 (2230454)	*MAB_1517c*	Probable O-methyltransferase Omt	
OP4 (396003)	*MAB_2649*	Putative membrane protein. MmpS family	
	*MAB_2650*	Putative membrane protein. MmpL family	
OP5 (2231454)	*MAB_4663*	Hypothetical protein	No IPS
OP6 (2231514)	*MAB_4791c*	Hypothetical protein	Twin-arginine translocation pathway. Signal sequence
OP7 (2229980)	*MAB_0086*	Taurine catabolism dioxygenase	
OP8 (395598)	*MAB_0734*	Hypothetical protein	Leukocidin/porin MspA superfamily (036435)
OP9 (396109)	*MAB_3132c*	Membrane protein	No IPS
	*MAB_3133c*	Hemin transporter	
	*MAB_3134c*	Transcriptional regulator	
OP10 (2231416)	*MAB_4509c*	Hypothetical protein	No IPS
OP11 (22311420)	*MAB_4532c*	Hypothetical protein	N-acetyltransferase Eis (016181)

^a^Mabs gene: *Mycobacterium abscessus* gene (accession number NC_010397.1).

^b^IPS: InterProScan protein signature.

**Fig 5:**
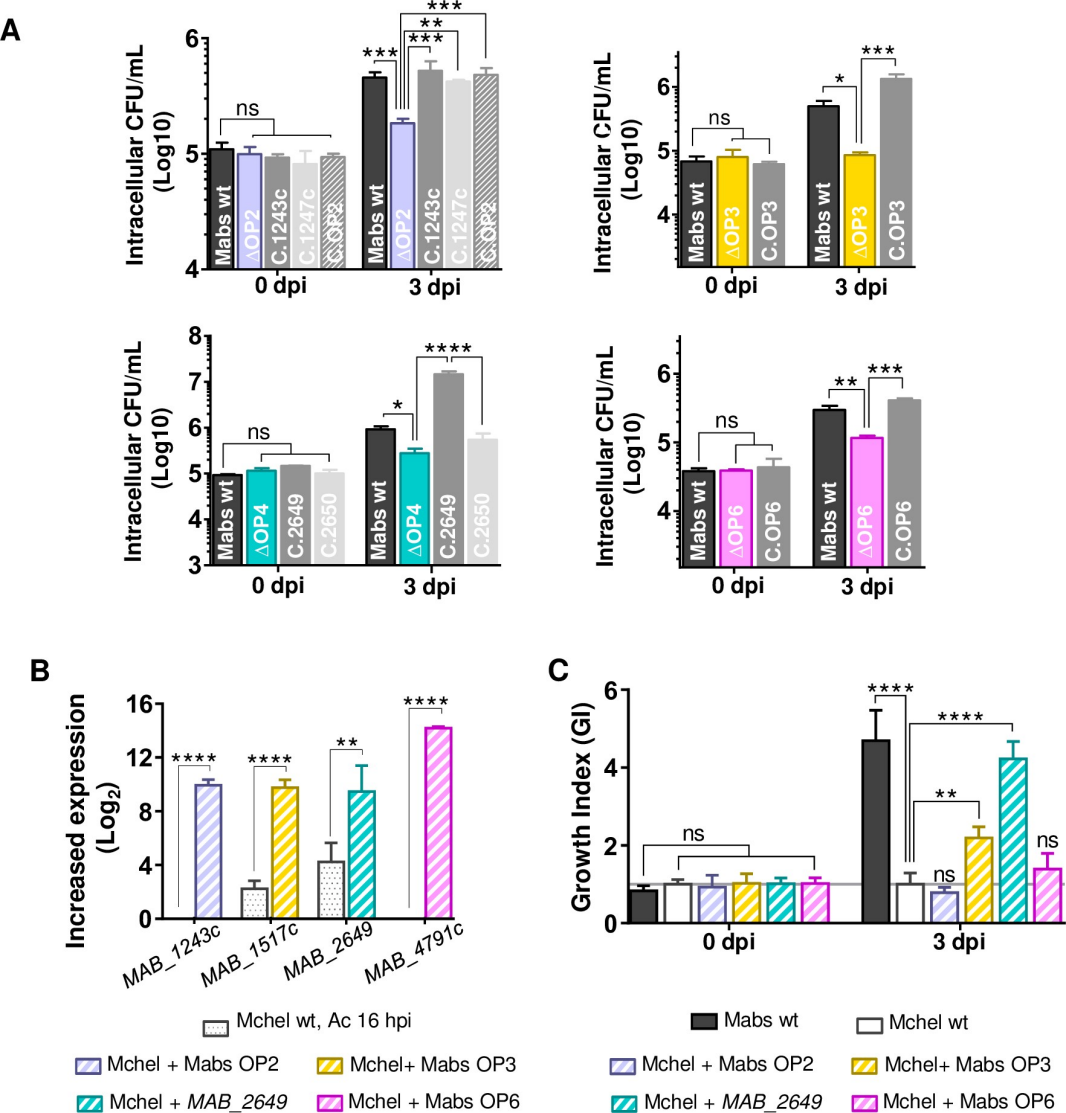
Comparative transcriptomic analyses allow identifying genes required for *M*. *abscessus* survival in amoebae and Mϕ. **(**A) Intracellular survival of 4 selected KO (ΔOP) and complemented strains in Mϕ. Mϕ were infected at 10 MOI and colony forming units (CFU) tests were performed 0 and 3 dpi. (B**)** Over-expression *M*. *abscessus* selected virulence genes in *M*. *chelonae*. FC values from *M*. *chelonae* transcriptome in Ac 16 hpi are compared to FC value mid-log phase cultures of *M*. *chelonae* strains overexpressing *M*. *abscessus* genes. (C**)** *M*. *chelonae* wt and *M*. *chelonae* overexpressing *M*. *abscessus* selected virulence genes replication in Mϕ relative to *M*. *abscessus*. Cells were infected at 10 MOI and colony forming units (CFU) tests were performed 0 and 3 dpi. The relative growth of each strain as compared to *M*. *abscessus* wt (Growth Index, GI) is given. All experiments were repeated twice or more, in duplicates (A) or triplicates (B). Statistical analyses were performed with GraphPad PRISM6. Histograms with error bars represent means ± SD. Differences between means were analyzed by ANOVA and the Tukey post-test allowing multiple comparisons to be performed. ns = non-significant. * *p*<0.05, ** *p*<0.01, *** *p*<0.001, **** *p*<0.0001.

ΔOP1 to ΔOP6 strains were evaluated for their intracellular multiplication in Ac and in Mϕ (**S3A Fig**). All mutants were attenuated in Ac and Mϕ, except one (ΔOP5),(**S3A Fig**). All mutant strains had similar growth *in vitro* compared to the wild type strain (**S3B Fig**). Among the genes selected for mutagenesis, several (OP2 and 6) are absent from *M*. *chelonae*, or if present in the *M*. *chelonae* genome they were at least four times less induced (OP3 and OP4) in Ac. To demonstrate the contribution of those genes to *M*. *abscessus* virulence in macrophages, we first complemented the corresponding KO strains (ΔOP2, 3, 4 and 6). All strains recovered the wt phenotype except for the OP4 gene *MAB_2650* potentially encoding an MmpL (**Fig 5A**). We further analyzed their contribution towards intracellular survival by overexpressing *M*. *abscessus* OP2, 3, 6 and *MAB_2649* genes in *M*. *chelonae* (**Fig 5B**), whose growth is restricted in Mϕ in comparison to *M*. *abscessus* [27,37]. Only the overexpression of *M*. *abscessus* OP3 and OP4 (*MAB_2649*) increased *M*. *chelonae* survival in Mϕ (**Fig 5C**). By comparison, no increase in *M*. *chelonae* intracellular survival was observed when overexpressing OP2 and OP6 (**Fig 5C**).

### *M*. *abscessus* highly-induced genes in Mϕ

**Fig 6:**
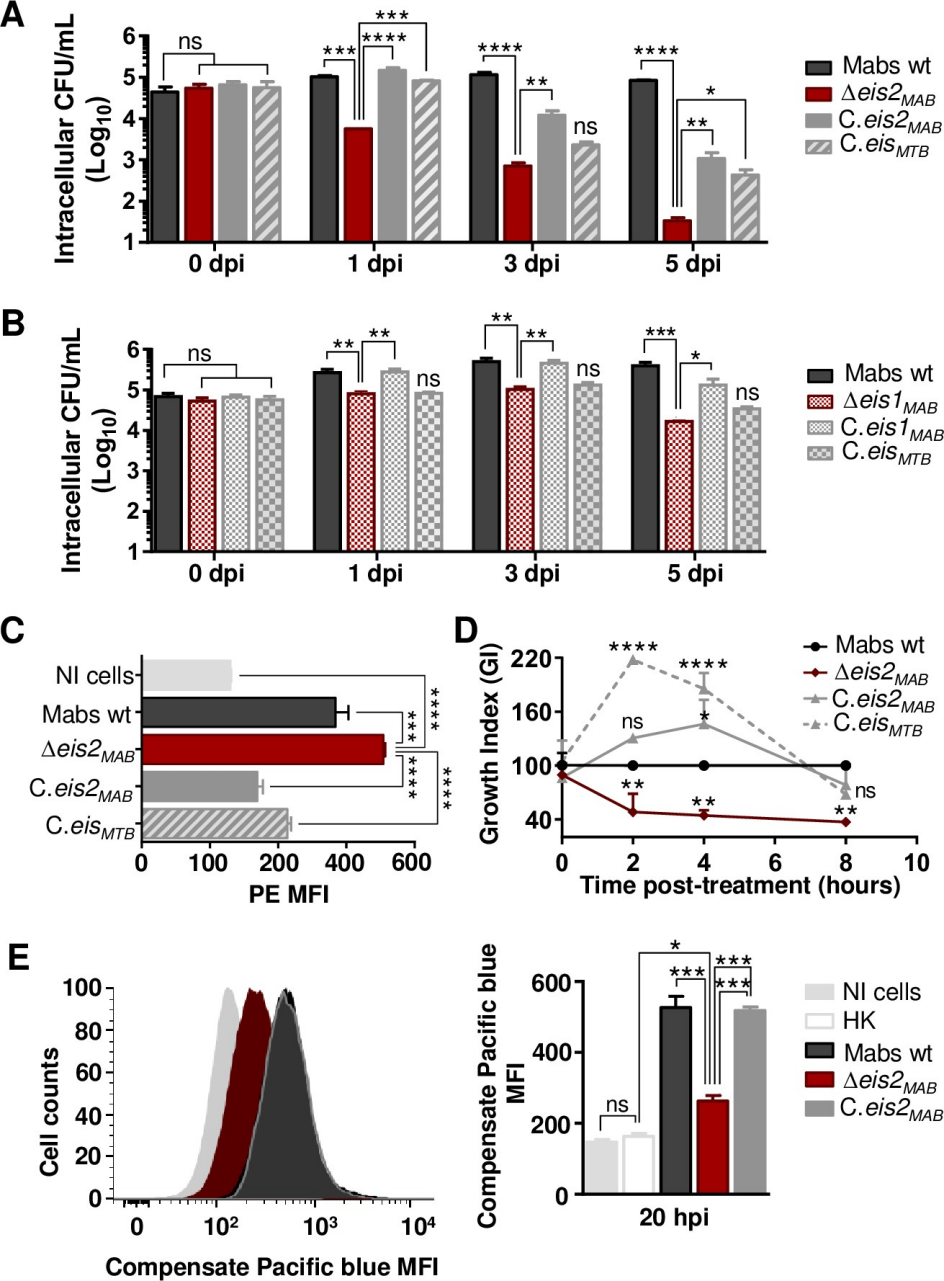
*M*. *abscessus eis2* gene is essential for survival in Mϕ and shares functions with *M*. *tuberculosis eis* conversely to *M*. *abscessus eis1*. **(**A) Intracellular survival of *M*. *abscessus eis2* KO strain (Δ*eis2*_*MAB*_) and complementation in Mϕ. (B**)** Intracellular survival of *M*. *abscessus eis1* KO strain (Δ*eis1*_*MAB*_) and complementation in Mϕ. Mϕ were infected at 10 MOI and colony forming unit (CFU) tests were performed at several times post-infection (0, 1, 3 and 5 dpi). (C**)** Control of ROS production by *M*. *abscessus* Eis2. ROS production by Mϕ was assessed by flow cytometry with the Mitosox Red kit, 15 min post-infection at 50 MOI. (D**)** Sensitivity of *M*. *abscessus eis2* KO strain to hydrogen peroxide (H_2_O_2_). Sensitivity to H_2_O_2_ was assessed by incubated bacterial cultures with H_2_O_2_ 20 μM during 8 h. The amount of survival cells was determined by performing CFU tests at several hours post-infection (2, 4, 8 hpi). **(**E**)** Control of phagosomal rupture by *M*. *abscessus Eis2*. Phagosomal rupture was assessed by performing a FRET analysis as previously described [73]. Results are depicted as signal overlays per group with 1,000,000 events per condition acquired in not infected cells (NI cells), Heat killed *M*. *abscessus* (HK), wild-type *M*. *abscessus* (Mabs wt), KO strains (Δ*eis2*_*MAB*_), KO strains complemented with *eis2*_*MAB*_ (C.*eis2*_*MAB*_). All experiments were repeated twice or more in triplicates. Statistical analyses were performed with GraphPad PRISM6. Histograms with error bars represent means ± SD. Differences between means were analyzed by ANOVA and the Tukey post-test allowing multiple comparisons to be performed. ns = non-significant. * *p*<0.05, ** *p*<0.01, *** *p*<0.001, **** *p*<0.0001.

The observed defect in intracellular survival was noticed for all mutants from OP7 to OP11 (**S3A Fig**), the ΔOP11 mutant was particularly attenuated (GI<0.1). OP11 (*MAB_4532c*) KO strain tended to be eliminated by Mϕ while the growth of the KO strain was similar to the WT growth *in vitro* (**S3B Fig**). When complementing ΔOP11 strain with *MAB_4532c*, we restored the wt phenotype (**Fig 6A**). *MAB_4532c* encodes an Eis N-acetyl transferase protein, according to a motif analysis (InterProScan). Of interest, *M*. *abscessus* contains two *eis* genes, named *eis1*_*MAB*_ (*MAB_4124*) and *eis2*_*MAB*_ (*MAB_4532c*), whereas *M*. *tuberculosis* possesses a single *eis*_*MTB*_ gene (*Rv2416c*), *eis1*_*MAB*_ (*MAB_4124*) being the closest homolog by Bidirectional Best Hit (BBH) search. No conservation of synteny was observed between the respective genomic regions in *M*. *abscessus* and *M*. *tuberculosis* (**S4A Fig**). In contrast, the *eis2*_*MAB*_ locus shows some similarity and conservation with the *M*. *tuberculosis mmpL11* locus, with inverted and syntenic conservation of groups of genes (**S4B Fig**). The *eis*_*MTB*_ locus is well-conserved in *M*. *abscessus* and corresponds to *MAB_1619*-*MAB_1627* and *MAB_1633*-*MAB_1637* regions (**S4C Fig**). Both *eis1*_*MAB*_ and *eis2*_*MAB*_ were found close to *mmpL* (brown arrows) and/or *mmpS* (orange arrow) genes (**S4 Fig**). In the *M*. *abscessus eis2* locus, an *mmpL* gene (*MAB_4529*) was conserved in *M*. *tuberculosis* corresponding to *mmpL11* (**S4B Fig**). To assess the function of *M*. *abscessus eis* genes, we first looked at the transcription and intracellular survival profiles and stated that unlike *eis2*_*MAB*_, *eis1*_*MAB*_ was suppressed inside Mϕ (**S5 Fig**) and less impaired in its intracellular survival (**Fig 6A and 6B**). Complementation of Δ*eis1*_*MAB*_ and Δ*eis2*_*MAB*_ with *eis1*_*MAB*_ or *eis2*_*MAB*,_ respectively, allowed the recovery of the intracellular survival for both mutants (**Fig 6A and 6B**). We also performed transcomplementation of the Δ*eis1*_*MAB*_ and Δ*eis2*_*MAB*_ KO strains with the *M*. *tuberculosis eis* (*eis*_*MTB*_) variant. Of note, complementation of the mutants with the *eis*_*MTB*_ gene allowed only partial restoration of the intracellular replicative phenotype for the Δ*eis2*_*MAB*_ mutant, but no restoration was observed for Δ*eis1*_*MAB*_ mutant (**Fig 6A and 6B**). Similar behaviors regarding apoptosis, necrosis, autophagy and phagosomal acidification were observed when comparing the wt *M*. *abscessus* strain with the Δ*eis2*_*MAB*_ mutant (**S6 Fig**). However, two major differences were observed. First, infection of Mϕ with the Δ*eis2*_*MAB*_ strain (at a MOI of 50) was associated with higher production of ROS by the cells and loss of *eis2*_*MAB*_ also sensitized *M*. *abscessus* to ROS and notably to H_2_O_2_ (**Fig 6C and 6D**). Secondly, the Δ*eis2*_*MAB*_ mutant was unable to damage the phagosomal membrane and to provoke phagosome-cytosol contact as compared to the wt and complemented *M*. *abscessus* strains (**Fig 6E**).

## Discussion

The main objective of this work was to understand the genetic and molecular basis for the ability of smooth *M*. *abscessus* strains to withstand and survive in eukaryotic phagocytic cells. We focused our experiments on S variants as from previous work we know that this morphotype, in contrast to R variants, represents the most infectious and intracellular forms of *M*. *abscessus* strains, whereas the R form rather represents the extracellular versions of the bacteria [38]. Transcriptome analyses of *M*. *abscessus* 43S and *M*. *chelonae* strains infecting amoeba and Mϕ, respectively, revealed genes strongly induced during infection of phagocytes. Selected KO mutants constructed in *M*. *abscessus* CIP 104536T on the basis of the results from transcriptomic analyses of *M*. *abscessus* 43S allowed us to confirm that the genes, which were highlighted by our transcriptomic approaches were indeed required for mycobacterial survival in phagocytes, even in a different genetic *M*. *abscessus* strain background. These results are complementary to a previous *Tn M*. *abscessus* library viability screen in Ac, which had identified two other intracellular virulence factors, namely the type VII secretion system ESX-4 and the lipid transport protein MmpL8_MAB_ [29,39]. The intracellular defects of strains that were deleted for genes highly induced in Ac (induced at least four times more compared to the intra-macrophagic transcriptome of *M*. *abscessus* and to the intra-Ac transcriptome of *M*. *chelonae* (OP1 to OP6)), suggest that the transcriptomic changes observed following a co-culture in amoebae reflected the response of *M*. *abscessus* in Mϕ. These results are in agreement with previous findings, suggesting that co-culturing of *M*. *abscessus* in amoebae enhances the virulence of *M*. *abscessus* in subsequent mouse infection experiments, likely through the induction of the phospholipase C-encoding *plc* virulence gene [27].

Most of the 6 loci (OP1 to OP6), studied, encode for hypothetical proteins, with the exception of the *MAB_1517c* gene that encodes a probable O-methyltransferase (OP3), and the *MAB_2649* and *MAB_2650* genes encoding MmpS and MmpL mycobacterial membrane proteins, respectively (OP4) (**Table 2**) [40].

At the *M*. *abscessus* OP2 locus, that mainly comprises genes of unknown function, we used a motif analysis to identify an ABC transporter, FecCD/TroCD-like in the MAB_1243c protein and an alkaline shock protein Asp23 in the MAB_1247c protein. A motif analysis performed on the *M*. *abscessus* OP6 locus shows that *MAB_4791c* encodes a protein implicated in the twin-arginine translocation pathway (see below).

Of note, it was also found that the over-induction of *MAB_2649* and *MAB_1517c* in *M*. *chelonae* favors its replication in Mϕ, suggesting that the high induction of these two genes in amoeba may trigger *M*. *abscessus* virulence.

Finally, one of the most striking findings of our work, is the essential role of the *eis2*_*Mab*_ gene in early resistance to the microbicidal action of Mϕ, via phagosomal membrane damage and cytosol contact, that might favor the intracellular survival of *M*. *abscessus*. Although *M*. *abscessus* possesses two *Eis* genes, there is no redundancy in their respective functions; the *eis1*_*Mab*_ mutant presented a similar behavior to the wt strain in Mϕ, with only the loss of a log_10_ CFU at 5 dpi, compared to the quasi-total clearance of the *eis2*_*Mab*_ mutant in Mϕ. Despite higher genomic identity between *eis1*_*MAB*_ and *eis*_*MTB*_, the restoration of the phenotype when complemented with *eis*_*MTB*_ was observed only for the *eis2*_*MAB*_ mutant, demonstrating the similar role of *eis2*_*MAB*_ to what is described for *eis*_*MTB*_ in virulence. However the deletion of this gene in *M*. *abscessus* is more deleterious for the bacterium in Mϕ compared to the deletion of *eis*_*MTB*_ [41,42]_._ E*is*_*MTB*_ has been described as being important for *M*. *tuberculosis* survival inside Mϕ by controlling host cell apoptosis, autophagy, ROS production and innate immune defenses [43]. As also observed in *M*. *tuberculosis* [43], increasing the MOI (to 50) revealed further differences between *eis2*_*MAB*_ KO and wt strains with regard to resistance to oxidized derivatives; however, this bacterial-load effect has not yet been observed for cell death mechanisms.

One of the peculiarities of the locus *eis2*_*MAB*_ is that it shows similarities with a genomic region of *M*. *tuberculosis*, within which is also located the gene *mmpL11*. The potential counterpart in *M*. *abscessus* would be *MAB_4529* (**S5 Fig**). Most of MmpL proteins are described as lipid transporters implicated in cell physiology and virulence [40,44]. *M*. *abscessus* has 27 MmpLs, twice as much as *M*. *tuberculosis* [40]. In *M*. *tuberculosis*, MmpL11 is implicated in heme iron acquisition [45] and transport of mycolic acid wax ester and long-chain triacylglycerols [46]. Three genes conserved in the *M*. *abscessus eis2* locus encode for proteins belonging to lipid transport and metabolism pathways (COG I), which suggests, together with the conservation seen with the *M*. *tuberculosis mmpL11* locus, that the *M*. *abscessus eis2* locus might also participate in cell wall biogenesis [47].

Transcriptomic analysis revealed that nine *M*. *abscessus* genes, whose orthologues in *M*. *tuberculosis* contribute to virulence, were highly induced during infection of Mϕ (**S2 Table**). Among their gene products, WhiB7 and DevR-DevS are implicated in stress sensing [48]. WhiB7, a Fe-S cluster protein, was shown to be induced in response to perturbation in amino-acid metabolism, under reducing intracellular state, iron depletion and increased temperatures [49]. The 20-fold increase of *M*. *abscessus whiB7* in Mϕ suggests that *M*. *abscessus* may undergo similar stresses in Mϕ as *M*. *tuberculosis*. The DevR response regulator of the histidine kinase DevS was also highly up-regulated. In *M*. *tuberculosis*, the *devR-devS* two-component system (also known as the DosR system) is activated in response to hypoxia [50]. Likewise, *M*. *abscessus MAB_2562c*, the orthologue of *Rv0081*, was induced 10-fold in Mϕ. A putative orthologue (*MAB_1409c*) of the dormancy response gene *Rv1258c* was also strongly induced in intra-macrophagic *M*. *abscessu*s. The conserved alpha-ketoglutarate-dependent dioxygenase AlkB-encoding gene is thought to be involved in fatty acid metabolism, or in protection against DNA methylation. The *aspC* gene was induced 8-fold; AspC mediates nitrogen transfer from aspartate to glutamate, which in turn, together with glutamine, provides nitrogen to most of the biosynthesis pathways. This is thought to be essential in *M*. *tuberculosis* [51], while aspartate is required for mycobacterial virulence [52]. *M*. *abscessus katA* gene, which is conserved in *M*. *avium* and *Listeria monocytogenes*, is a catalase that degrades H_2_O_2_ into water and oxygen in a single reaction. Such a reaction, enabling resistance to oxidative metabolites, may be an important mechanism of bacillary survival within the host phagocyte [53]. *M*. *abscessus eamA (MAB_0677c)*, which is thought to encode a drug/metabolite transporter, was induced in Mϕ. Two additional genes (*MAB_3762* and *MAB_*3180) encoding proteins with an EamA domain were also highly induced. Finally, at the molecular function level, it appears that six of the most highly induced genes in *M*. *abscessus* in Mϕ encode acyl or N-acetyl transferase proteins playing a role in post-translational modifications.

The *M*. *abscessus* transcriptomes’ comparison in Ac or Mϕ allowed differences in metabolic adaptations to be highlighted. In Mϕ, *M*. *abscessus* enters a slow replicative stage, and activates the detoxification and protein secretion pathways. By comparison, in amoebae *M*. *abscessus* switches on protein synthesis, lipid transport and metabolism, transcription of genes involved in post-translational modifications (PTM), protein turnover and chaperones (COG O), reflecting a more active and replicative behavior as compared to a more persistent state in Mϕ. Actually, cell wall biogenesis including peptidoglycan and glycosaminoglycan biosynthetic processes were down-regulated in Mϕ. Similarly, *mtrA*, *phoP* and *devR* were differently regulated, with only *devR* up-regulated in Mϕ, confirming the switch towards a slow growth stage for *M*. *abscessus* in Mϕ.

Over-representation of the COG O (post-translational modification, protein turnover, molecular chaperone) category in *M*. *abscessus* infecting Ac indicates that *M*. *abscessus* may alter cellular processes during its interactions with host cells via PTM, as described in various pathogens [54–56]. Protein turnover does not only help in clearing of old proteins but also aids a fast adaptation to nutrient poor environments [57]. Molecular chaperones help pathogens override unfavorable conditions found in the host such as heat shock, oxidative and acid stresses [58]. They also contribute to the inhibition of lysosomal fusion and favor bacterial growth [58]. Molecular chaperones may therefore form a first line of defense and help consolidate pathogen virulence. Thus, over-representation of the COG O during infection of amoebae might reflect specific intracellular cues, mycobacteria face from the early time points post infection on.

In Ac, the most enriched GO is adenine salvage (GO:0006168) (**Fig 2**). This GO represents any process that generates adenine from derivatives without any *de novo* synthesis. Mycobacteria are able to limit the synthesis of this high energy demanding nucleotide [59]. Mycobacteria are also capable of scavenging free nitrogenous bases from the medium [59]. Under conditions of low energy availability or rapid multiplication, the salvage pathway may then be the main source of maintaining the nucleotide pool [59].

Sulfur metabolism (GO:0000103), hydrogen sulfide (H_2_S) biosynthetic pathway (GO:0070814) and detoxification via Fe-S cluster assembly proteins (GO:0016226) in addition to polyamine transport (GO:0015846), were also enriched by *M*. *abscessus* in Ac. In its reduced form, sulfur is used in the biosynthesis of the amino acid cysteine that is one of the prime targets for reactive nitrogen intermediates [60]. Those pathways might play a key role in *M*. *abscessus* survival in phagocytic cells, since genes involved in the metabolism of sulfur have consistently been identified as up-regulated in conditions that mimic the intra-macrophagic environment and during Mϕ infection for *M*. *tuberculosis* [36]. As for polyamines (cadaverine, putrescine and spermidine), they are known to have pleiotropic effects on cells via: their interaction with nucleic acids; a role in bacterial virulence by allowing mycobacterial escape from the phagolysosome; toxin activity or protection from oxidative and acid stress has also been demonstrated [61].

In Mϕ, glycerol ether metabolic process, MEP pathway and L-proline biosynthetic processes were the most enriched. Glycerol ether metabolic process corresponds to glycerophospholipids-seminolipids-plasmecholine metabolism and cellular amide biosynthetic processes. The MEP pathway is required for isoprenoid precursor biosynthesis [62]. A wide variety of monoterpenes and diterpenes belong to isoprenoid classes which function as toxins, growth inhibitors, or other secondary metabolites [63]. Finally, proline has been reported as an important factor in the adaptation of mycobacteria to slow growth rate and hypoxia [64]. It is believed that the proline-utilization pathway protects mycobacterial cells by detoxifying methylglyoxal, a by-product of endogenous glycerol metabolism [64] that can damage DNA and proteins within cells. Up-regulation of base-excision repair suggests that intracellular mycobacteria undergo DNA damage. Protein folding was also enriched, as well as the type II secretion system, which was enriched by more than two-fold. This secretion system promotes the specific transport of folded periplasmic proteins across a dedicated channel in the outer membrane, and it facilitates both Sec and Tat pathways to secrete proteins into the periplasm. Potential roles for SecA1 and SecA2 in *M*. *tuberculosis* dormancy has been reported while the Tat pathway was shown to contribute to virulence in *Legionella pneumophila* for instance, by aiding secretion of Phospholipase C [65], a virulence factor conserved in *M*. *abscessus* [21].

Both Ac and Mϕ were sensed as a stressful environment by *M*. *abscessus*, evidenced by the up-regulation of genes known to be involved in multiple stress responses. Induction of low O_2_ and low NO response genes confirm that hypoxic environments are encountered by *M*. *abscessus* both in Ac and Mϕ.

In conclusion, our findings confirm that the amoeba-induced genes play a role in potentiating the subsequent survival of *M*. *abscessus* in Mϕ. Both environments have commonalities, in terms of metabolic switches, especially to withstand the host response. It is likely that through such preparation during its intra-amoebic life that *M*. *abscessus* is able to withstand the noxious Mϕ environment, especially due to selected genes whose role has been highlighted during this work. The multiple leads opened during this work must now be followed to complete this viewpoint of synergistic potentiation of virulence conferred by the amoeba to *M*. *abscessus*, including the ultimate mechanisms of manipulation of the host's defense systems as seen with other intracellular pathogens.

## Materials and methods

### Bacterial strains, plasmids and growth conditions

A clinical isolate of *M*. *abscessus* subspecies *massiliense* smooth variant (43S) and *M*. *chelonae* type strain CCUG 47445 were used for the RNAseq experiments. Gene deletions were performed with CIP 104536T type smooth strain of *M*. *abscessus* subspecies *abscessus*. Both *M*. *abscessus* CIP 104536T strain and *M*. *chelonae* CCUG 47445 type strains were used to perform *in vitro* survival and complementation tests, while gene deletion experiments were performed with *M*. *abscessus* CIP 104536T. *M*. *abscessus* and *M*. *chelonae* strains were routinely grown aerobically at 37°C and 32°C respectively, in Middlebrook 7H9 medium (Sigma-Aldrich) supplemented with 0.2% glycerol, 1% glucose, and 250 mg/L kanamycin (Thermo Fisher Scientific) when necessary, with 25 mg/L zeocin (ThermoFisher Scientific) for the knockout strains, and with 25 mg/L zeocin plus 250 mg/L hygromycin (InvivoGen) for complemented strains. *A*. *castellanii* (ATCC 30010) was grown at room temperature without CO_2_ in peptone-yeast-extract-glucose (PYG) broth for the amplification of the strain. Mouse Mϕ J774.2 (Sigma) were grown and used as described [38,66].

### Gene deletion and complementation

Deletion of genes was performed using the recombineering system as described previously [27,67]. Growth of the KO strains was checked by measuring the optical density of bacterial cultures in 7H9 medium supplemented with glycerol 0.2%. Complementation was performed after amplifying and cloning genes into the integrative plasmid pMVH361 as described [27].

### RNA isolation and RNA sequencing

Approximately 10^7^ cells were infected in 50 mL tubes, with low agitation, without CO_2_. Amoebae were infected at 100 MOI at 32°C in Page’s modified Neff’s amoeba saline (PAS) [68]. J774.2 Mϕ were infected at 50 MOI at 37°C in Dulbecco's Modified Eagle Medium (DMEM).

Cells were washed 3 times after 1 hour of infection and resuspended in medium supplemented with amikacin 250 μg/mL and incubated for 1 hour to eliminate extracellular bacteria. These relatively high MOI, employed for a limited time (1 h) were chosen to assure a sufficient infection rate of cells, as required for RNA sequencing. Three additional washes were performed and cells were resuspended in medium supplemented with amikacin 50 μg/mL for the rest of the infection. Amoebal cells were harvested 4 h and 16 hpi for intracellular *M*. *abscessus* RNA isolation and 16 hpi and for intracellular *M*. *chelonae* RNA isolation. Mϕ were harvested for intracellular *M*. *abscessus* RNA isolation 16 hpi. RNA isolation was performed as described [69]. Briefly, cells were lysed with a cold solution of guanidium thiocyanate (GTC), N-Lauryl-sarcosine, sodium citrate +/- Tween 80 plus β-mercaptoethanol. The lysates containing intracellular bacteria were collected, centrifuged and RNA was isolated from the bacterial pellets with TRIzol. The lysates were then transferred into 2 mL screw tubes containing zirconium beads and were conserved at -80°C for at least 1 day to allow inactivation of RNAses and cells dissolution. Bacteria cells were disrupted with a bead beater by performing to round at 6,500 rpm for 25 seconds, followed by one round at 6.500 rpm for 20 seconds. Two hundred μL of chloroform isoamyl alcohol were added and tubes were immediately mixed for 10 seconds. The mixture was centrifugated at 13,000 rpm for 15 minutes at 4°C. The RNA present in the upper phase was transferred to a fresh tube and precipitated by adding 0.8 volume of isopropanol. Tubes were inverted twice to allow precipitation and kept at -20°C for at least 2 hours. The precipitated RNA was then pelleted by centrifugation at 13,000 rpm for 30 min at 4°C. The pellet was washed with ethanol (70%) and centrifuged at 13,000 rpm for 10 min at 4°C. The washed pellet was air-dried, re-suspended in RNase-free water and stored at -80°C until cDNA library construction.

Control RNA was isolated from bacteria cells grown in amoeba (PAS buffer) or Mϕ co-culture medium (DMEM supplemented with 10% Fetal Bovine Serum respectively).

Biological replicates were prepared to allow statistical comparisons of infected and non-infected samples.

### RNA treatments prior to library preparation and library preparation

RNA samples were treated with DNases (AMBION) to remove DNA contaminants, purified with the RNA MEGAclear kit (ThermoFisher), and depleted of ribosomal RNA with the riboZero kit (Illumina). RNA (total, depleted, purified) was checked on the Bioanalyser system (Agilent) for its quality and integrity. cDNA libraries were prepared with samples displaying a RNA integrity number above 7. RNA concentrations were measured using the nanodrop spectrophotometer (Thermo Scientific) and the Qubit fluorometer (Invitrogen). Libraries were prepared with the TruSeq Stranded RNA LT prep kit cDNA synthesis, set A (Illumina) which consists in: (1) RNA fragmentation, (2) 1st strand cDNA synthesis (Reverse transcriptase and random primers), (3) 2^nd^ strand cDNA synthesis (removal of the RNA template and synthesis of a new strand with dUTP), (4) no end repair step, (5) adenylation of 3’ ends, (6) ligation of adapters and (7) enrichment of DNA fragments. Libraries were checked for concentration and quality on DNA chips with the Bioanalyzer Agilent. More precise and accurate quantification was performed with sensitive fluorescent-based quantitation assays ("Quant-It" assays kit and QuBit fluorometer, Invitrogen).

### NGS sequencing and data analyses

Sequencing and statistical analyses were performed in the Transcriptome and Epigenome platform (PF2) of the Pasteur Institute, Paris, France. The cDNA libraries for strains *M*. *abscessus* subsp. *massiliense* 43S and *M*. *chelonae* CCUG 47445 were prepared and sequenced on an Illumina HiSeq 2500 system by performing an SRM run (SR: Single Read, PE: Paired-end Reads, M: multiplexed samples) of 51 cycles with 7 index bases read. The quality of the sequencing was assessed with the external FastQC program (https://www.bioinformatics.babraham.ac.uk/projects/fastqc/). After the trimming of adapter sequences and low-quality reads with cutadapt version 1.11, reads were aligned with RefSeq assemblies (*M*. *abscessus* subsp. *massiliense* strain GO06 assembly (GCF_000277775.2); *M*. *chelonae* CCUG 47445 assembly (GCF_001632805.1), using the Bowtie software version 0.12.7 (http://bowtie-bio.sourceforge.net/index.shtml) with defaults parameters. Genes were counted using featureCounts 1.4.6-p3 from Subreads package (parameters: -g gene -t ID -s 1). Differential analysis of gene expression was performed using the R software (version 3.3.1) and the Bioconductor packages DESeq2 (version 1.12.3) [30] using the default parameters and statistical tests for differential expression were performed applying the independent filtering algorithm. A generalized linear model was set in order to test for the differential expression between the biological conditions. For each pairwise comparison, raw *p*-values were adjusted for multiple testing according to the Benjamini and Hochberg (BH) procedure [70] and genes with an adjusted *p*-value lower than 0.05 were considered differentially expressed. Gene orthologs of *M*. *massiliense* and *M*. *chelonae* genes in the genome of the *M*. *abscessus* subsp. *abscessus* CIP 104536T reference strain were determined by Bi-directionnal Best Hit (BBH) searches using the Opscan software (http://wwwabi.snv.jussieu.fr/public/opscan/). Differentially expressed genes assignment to COGs was performed using the COG automatic Classification from the MicroScope database [71]. The percentage assignments were compared by performing Fisher’s exact tests. GO enrichment analyses were performed with the R software topGO package (Bioconductor) [72]. Protein signatures were addressed using InterProScan tool (https://www.ebi.ac.uk/interpro/search/sequence-search).

### Quantitative real-time PCR (qRT-PCR)

qRT-PCR were performed with a CFX96 thermal cycler (Bio-Rad). Controls without reverse transcriptase were done on each RNA sample to rule out DNA contamination. The sigA gene was used as an internal control [27]. Each qRT-PCR was performed with three biological replicates.

### *In vitro* survival assays

Survival of strains in amoebae and J774.2 Mϕ were performed as previously described [39]. Survival tests of KO strains were performed in duplicates three times. Confirmation of attenuated phenotypes and complementation tests were performed in triplicates three times.

### Phagosome acidification and phagosomal escape assays

Phagosome acidification and phagosomal escape Fluorescence Energy Transfer ***(***FRET) assays were conducted in J774.2 Mϕ as previously described [38,73].

### Cell death, autophagy and ROS production assays

Mϕ death following infection with *M*. *abscessus* was assessed with the Dead Cell Apoptosis Kit with Annexin V FITC and PI for flow cytometry (ThermoFisher). Autophagy was assessed with the Premo Autophagy Tandem Sensor RFP-GFP-LC3B Kit (ThermoFisher). ROS production by J774.2 Mϕ was measured with the MitoSOX Red kit (ThermoFisher).

Infections were performed as previously described [39], at 50 MOI, except in the ROS production assay for which the cells were infected 15 min only.

### Bacterial sensitivity to H_2_O_2_

Sensitivity to H_2_O_2_ was assessed by culturing the bacteria in 7H9 medium supplemented with glycerol 0,1% and H_2_0_2_ 3% (Laboratoires Gilbert) (20 μM). CFU tests were performed at different times post-treatment (2 h, 4 h, 8 h) to determine the number of viable bacteria compared to the wt strain (Growth Index).

## Supporting information

S1 Fig DESeq2 statistical analyses. **A.** *M*. *abscessus* transcriptomes in *A*. *castellanii* 4 and 16 hpi. **B.** *M*. *abscessus* transcriptome in macrophages 16 hpi**. C.** *M*. *chelonae* transcriptome in *A*. *castellanii* 16 hpi. Hierarchical clustering of raw data (left panel) and transcriptome heatmaps (right panel) were depicted. Clustered were indicated by red and blue circles corresponding to raw data from intracellular bacteria and planktonic bacteria respectively. Hatched and filled circles correspond to 4 h and 16 h (co)-cultures respectively. Change in gene expression were depicted on the heatmap in a white to blue scale for repressed genes and a white to red scale for induced genes, the white color representing no change in gene expression.
(TIF)

S2 Fig Comparison of *M. abscessus* transcriptomes in *A. castellanii* and in macrophages according to differentially expressed genes fold change. **A.** Differentially expressed genes (DEGs) from comparisons of co-cultures with *A*. *castellanii* (Ac) and macrophages (Mϕ) relative to planktonic growth were categorized according to their fold change (FC) expressed in Log_2_. Low DEGs depict a FC < |2|, Med DEGs depict a FC between |2| and |4| and High DEGs depict a FC > than |4|. **B.** Ratio of UP DEGs over DOWN DEGs.
(TIF)

S3 Fig Verification of knockout (KO) strains growth in culture medium and contribution to virulence in Mϕ. **A.** Intracellular survival of KO strains (ΔOP) in *A*. *castellanii* (Ac) and macrophages (Mϕ). Cells were infected at 10 MOI and colony forming units (CFU) tests were performed 0 and 3 dpi. The relative growth of each strain as compared to *M*. *abscessus* wt (Growth Index, GI) is given. **B.** KO strains growth in culture medium. The strains were cultured in 7H9 medium supplemented with glycerol 0.2% for seven days. Growth curves were obtained by measuring the cultures optical density each day. Experiments were repeated three times in triplicates. Statistical analyses were performed with GraphPad PRISM6. Histograms with error bars represent means ± SD. Differences between means were analyzed by ANOVA and the Tukey post-test allowing multiple comparisons to be performed. ns = non-significant, * *p*<0.05, ** *p*<0.01, *** *p*<0.001, **** *p*<0.0001.
(TIF)

S4 Fig Conservation of *M. abscessus eis* loci in *Mycobacterium tuberculosis* and vice versa. **A.** Conservation of *M*. *abscessus eis1* locus in *M*. *tuberculosis*. **B.** Conservation of *M*. *abscessus eis2* locus in *M*. *tuberculosis*. **C.** Conservation of *M*. *tuberculosis eis* locus in *M*. *abscessus*. Bidirectional Best Hit (BBH) search was performed between *M*. *abscessus* and *M*. *tuberculosis* genomes with the Opscan software. BBHs were depicted by arrows filled with red, brown or orange. Brown arrows correspond to MmpL-encoding genes. Orange arrows correspond to MmpS-encoding genes. Greys bands link genes or groups of genes conserved in the two species.
(TIF)

S5 Fig Expression of *M. abscessus eis* genes in Mϕ 4 and 16 hpi. *Eis1*_*MAB*_ (left panel) and *eis2*_*MAB*_ (right panel) expression in Mϕ was measured twice in triplicates by quantitative-real time PCR by normalization with *sigA* housekeeping gene.
(TIF)

S6 Fig Intracellular phenotypes uncontrolled by *M. abscessus eis2* genes. **A.** Cell death. Mϕ death following infection with *M*. *abscessus* was assessed with the Dead Cell Apoptosis Kit with Annexin V FITC and PI for flow cytometry. **B.** Cell autophagy was measured Premo Autophagy Tandem Sensor RFP-GFP-LC3B Kit. At least 40 cells per condition were analyzed by confocal microscopy. To assess the number of autophagic particles per cell, cell nucleus was stained with Hoechst 33342 (blue spots). Representative images of autophagic particles were given. Stained cells with the premo-autophagy kit were either non-infected (NI cells) or infected with *M*. *abscessus* strains (Mabs wt and Δ*eis2*_*MAB*_) or treated with chloroquine 30 μM for 48 h (+chloro) inhibiting autophagy or with HBSS solution for 2 hours (+HBSS) inducing autophagy. Autophagic particles are represented in red. Stained cells with the premo-autophagy kit were either non-infected (NI cells) or infected with *M*. *abscessus* strains (Mabs wt and Δ*eis2*_*MAB*_) or treated with chloroquine 30 μM for 48 h (+chloro) inhibiting autophagy or with HBSS solution for 2 hours (+HBSS) inducing autophagy. Acidification of autophagosomes was determined by dividing GFP (sensitive to acidic pH) over RFP (no sensitive to acidic pH) fluorescence intensity. The number of autophagic particles per cell and acidification of autophagosomes were determined with the Fiji software. **C.** Phagosomal acidification was assessed as previously described [38]. Mϕ were infected at 10 (C) or 30 MOI (A and B). Histograms with error bars represent means ± SD. Differences between means were analyzed by ANOVA and the Tukey post-test allowing multiple comparisons to be performed. ns = non-significant. * *p*<0.05, ** *p*<0.01, *** *p*<0.001, **** *p*<0.0001.
(TIF)

S1 Table Differentially expressed genes identified with the *DEseq2* package. (DOCX)

S2 Table List of *M*. *abscessus* genes highly induced in Ac only. (DOCX)

S3 Table List of *M*. *abscessus* genes highly induced in Mϕ or Ac 16 hpi. (DOCX)
