# Supplementary figures and images for: Mycobacterium abscessus virulence traits unraveled by transcriptomic profiling in amoeba and macrophages
Source: PLoS Pathog. 2019 Nov 8;15(11):e1008069. doi: 10.1371/journal.ppat.1008069 (PMC6839843; doi:10.1371/journal.ppat.1008069)

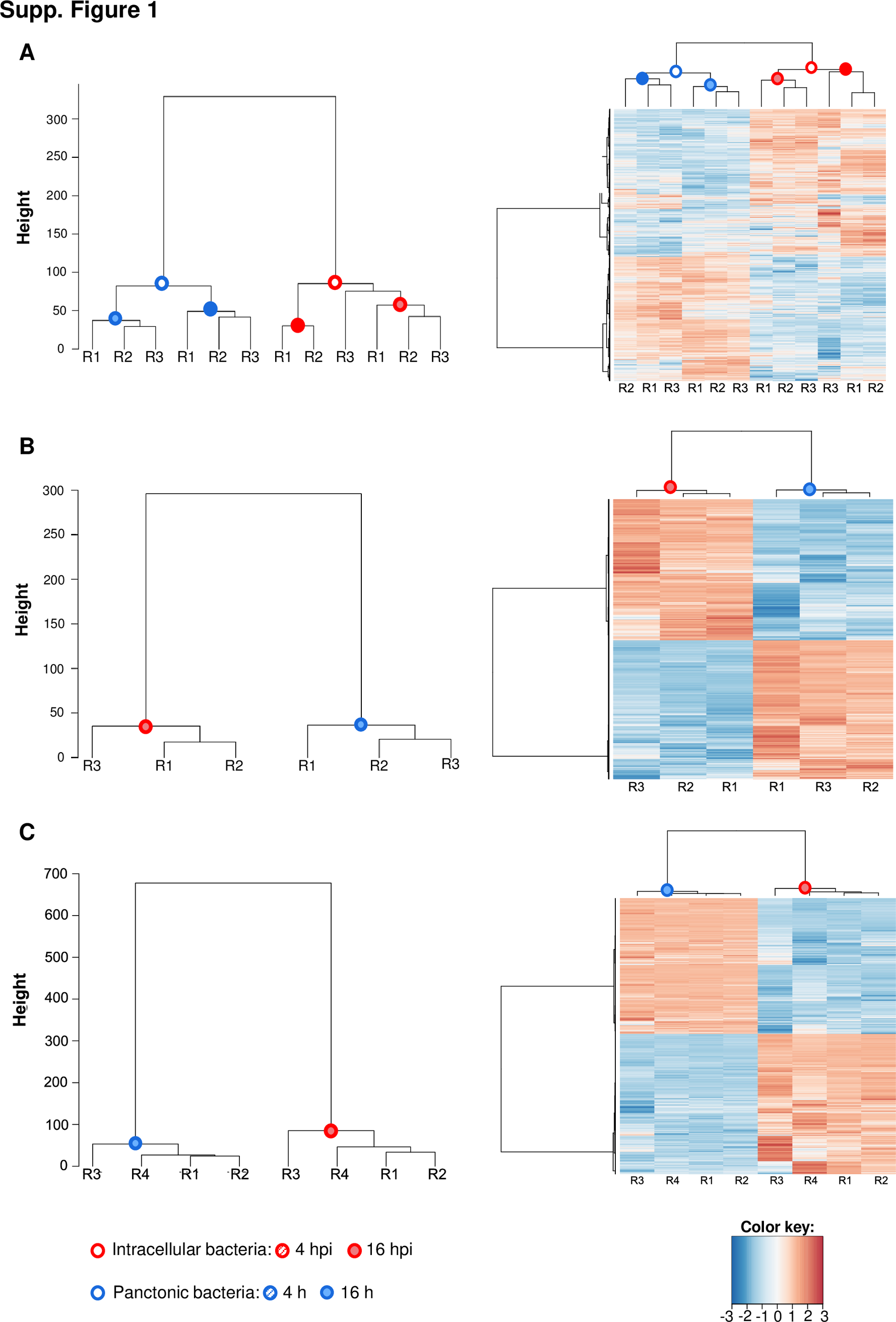

Supplement: S1 Fig — A. M. abscessus transcriptomes in A. castellanii 4 and 16 hpi. B. M. abscessus transcriptome in macrophages 16 hpi. C. M. chelonae transcriptome in A. castellanii 16 hpi. Hierarchical clustering of raw data (left panel) and transcriptome heatmaps (right panel) were depicted. Clustered were indicated by red and blue circles corresponding to raw data from intracellular bacteria and planktonic bacteria respectively. Hatched and filled circles correspond to 4 h and 16 h (co)-cultures respectively. Change in gene expression were depicted on the heatmap in a white to blue scale for repressed genes and a white to red scale for induced genes, the white color representing no change in gene expression. (TIF) [file ppat.1008069.s001.tif]

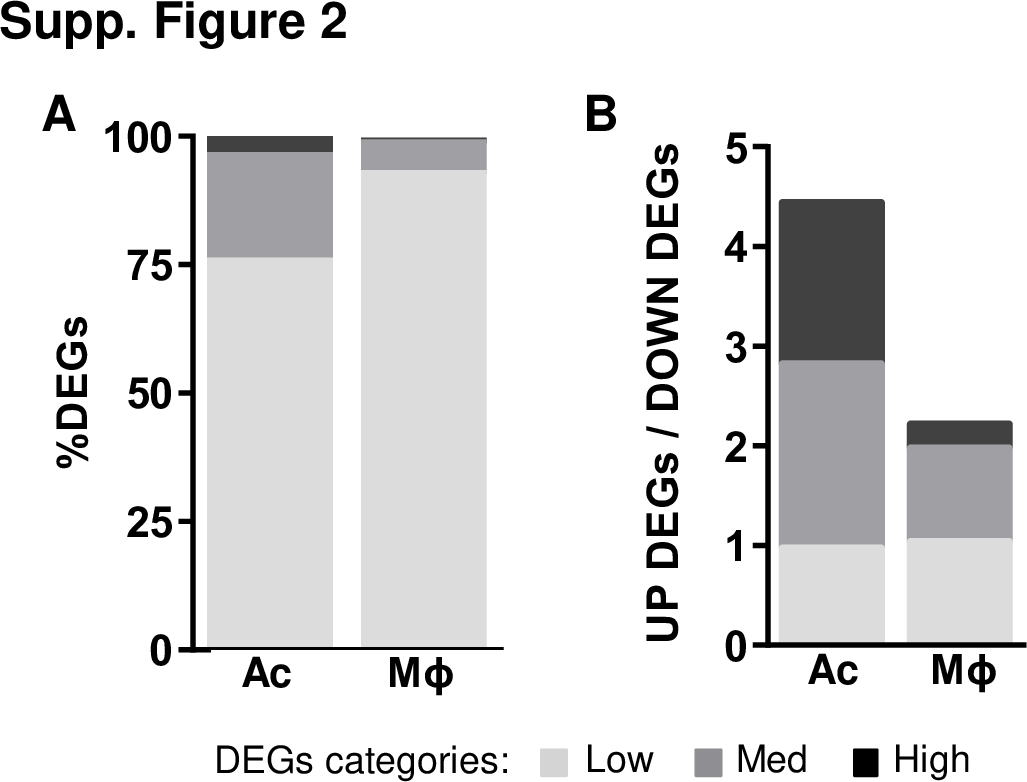

Supplement: S2 Fig — A. Differentially expressed genes (DEGs) from comparisons of co-cultures with A. castellanii (Ac) and macrophages (Mϕ) relative to planktonic growth were categorized according to their fold change (FC) expressed in Log2. Low DEGs depict a FC < |2|, Med DEGs depict a FC between |2| and |4| and High DEGs depict a FC > than |4|. B. Ratio of UP DEGs over DOWN DEGs. (TIF) [file ppat.1008069.s002.tif]

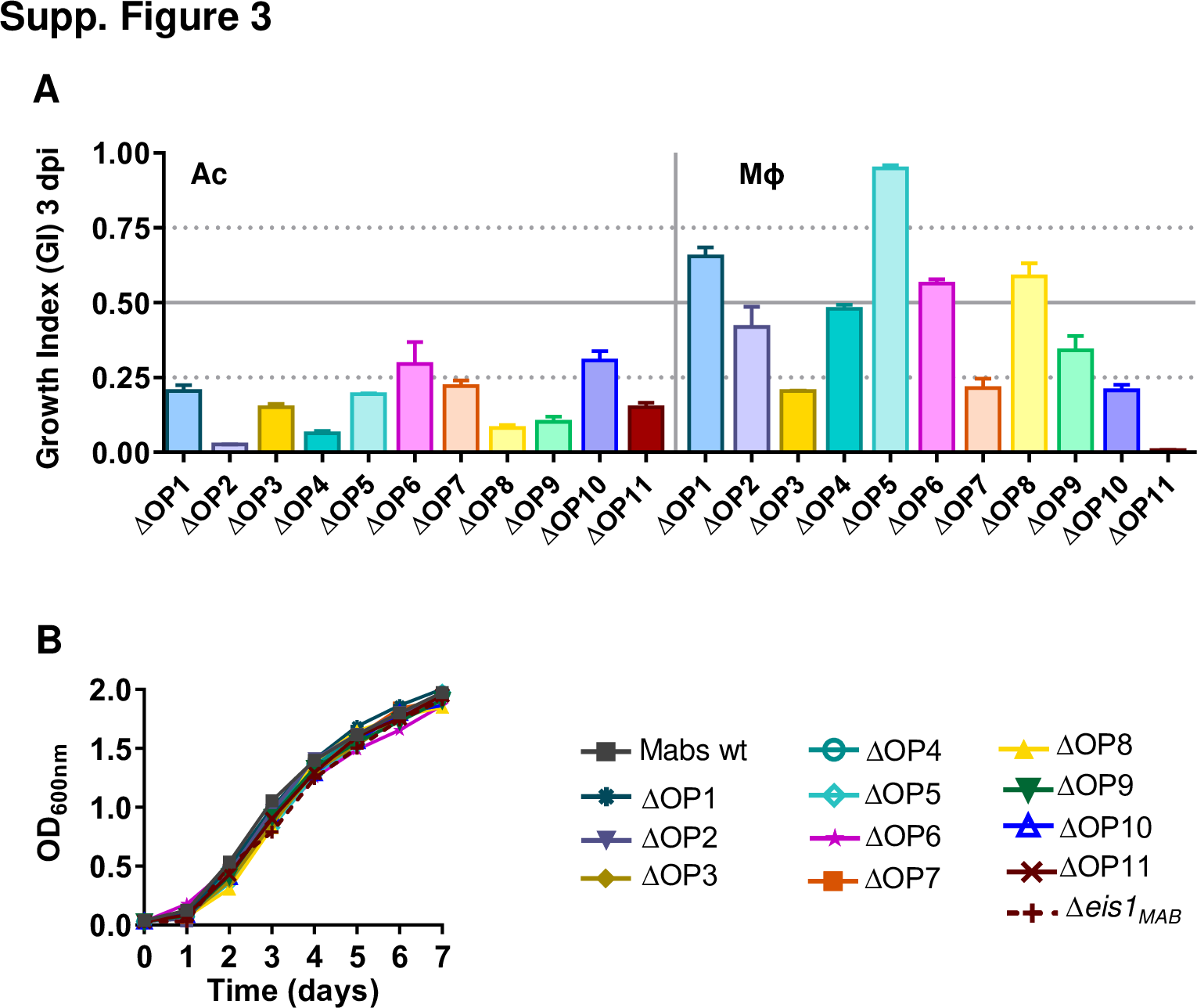

Supplement: S3 Fig — A. Intracellular survival of KO strains (ΔOP) in A. castellanii (Ac) and macrophages (Mϕ). Cells were infected at 10 MOI and colony forming units (CFU) tests were performed 0 and 3 dpi. The relative growth of each strain as compared to M. abscessus wt (Growth Index, GI) is given. B. KO strains growth in culture medium. The strains were cultured in 7H9 medium supplemented with glycerol 0.2% for seven days. Growth curves were obtained by measuring the cultures optical density each day. Experiments were repeated three times in triplicates. Statistical analyses were performed with GraphPad PRISM6. Histograms with error bars represent means ± SD. Differences between means were analyzed by ANOVA and the Tukey post-test allowing multiple comparisons to be performed. ns = non-significant, * p<0.05, ** p<0.01, *** p<0.001, **** p<0.0001. (TIF) [file ppat.1008069.s003.tif]

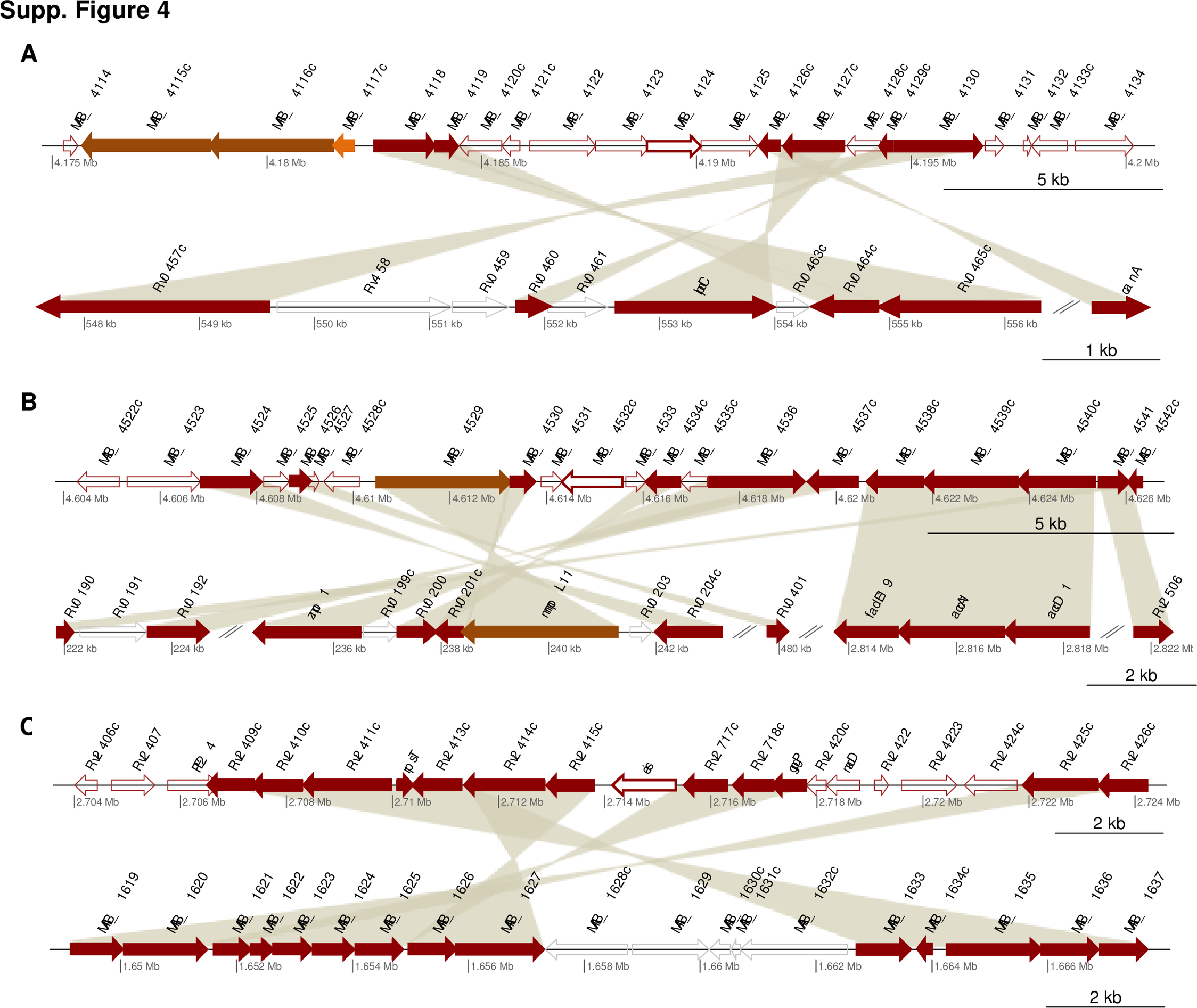

Supplement: S4 Fig — A. Conservation of M. abscessus eis1 locus in M. tuberculosis. B. Conservation of M. abscessus eis2 locus in M. tuberculosis. C. Conservation of M. tuberculosis eis locus in M. abscessus. Bidirectional Best Hit (BBH) search was performed between M. abscessus and M. tuberculosis genomes with the Opscan software. BBHs were depicted by arrows filled with red, brown or orange. Brown arrows correspond to MmpL-encoding genes. Orange arrows correspond to MmpS-encoding genes. Greys bands link genes or groups of genes conserved in the two species. (TIF) [file ppat.1008069.s004.tif]

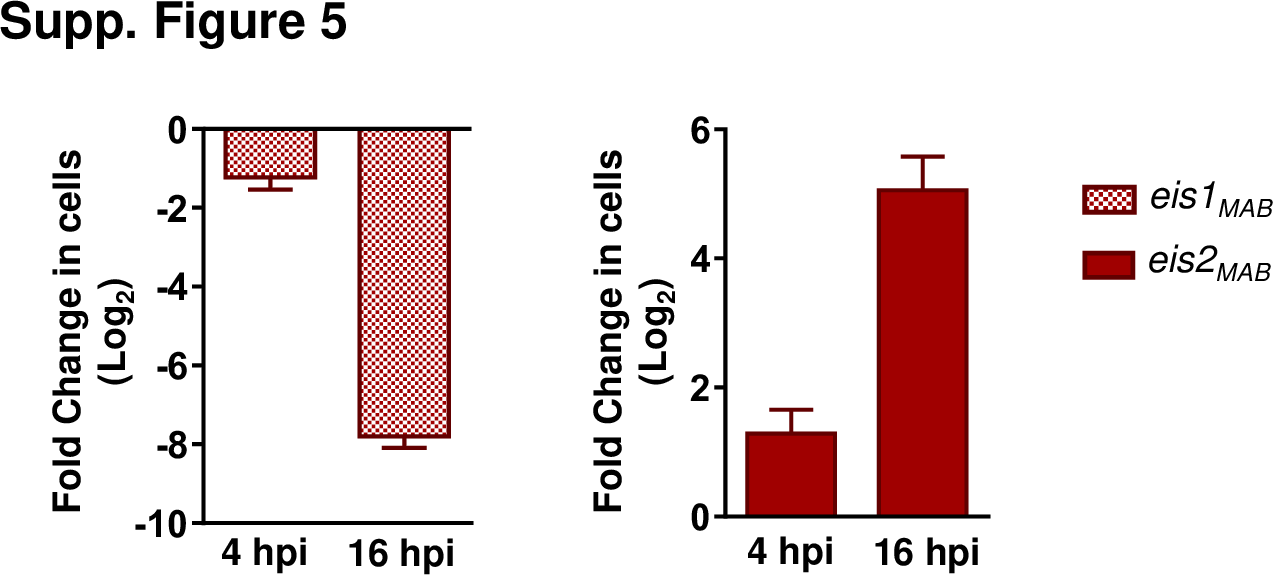

Supplement: S5 Fig — Eis1MAB (left panel) and eis2MAB (right panel) expression in Mϕ was measured twice in triplicates by quantitative-real time PCR by normalization with sigA housekeeping gene. (TIF) [file ppat.1008069.s005.tif]

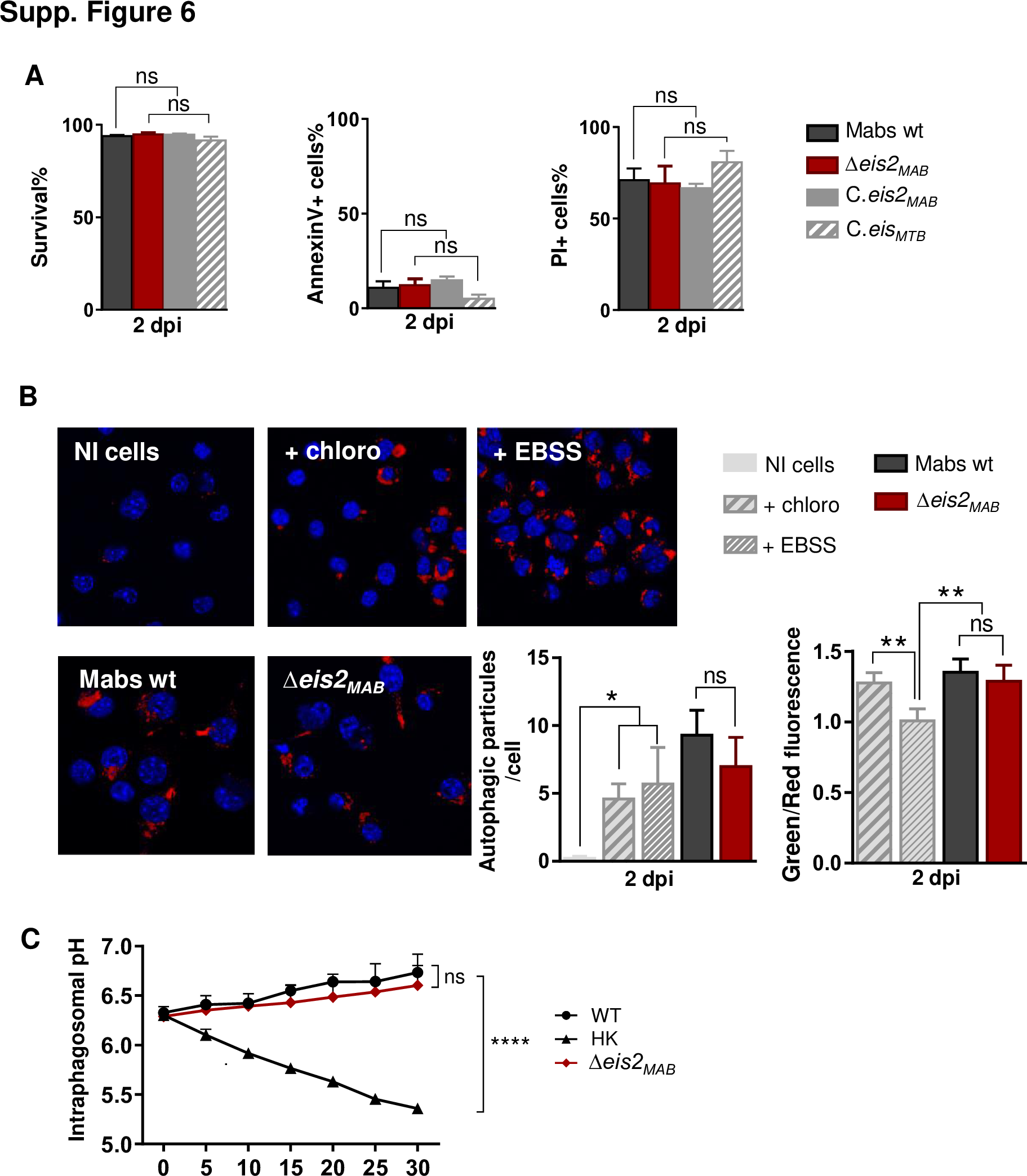

Supplement: S6 Fig — A. Cell death. Mϕ death following infection with M. abscessus was assessed with the Dead Cell Apoptosis Kit with Annexin V FITC and PI for flow cytometry. B. Cell autophagy was measured Premo Autophagy Tandem Sensor RFP-GFP-LC3B Kit. At least 40 cells per condition were analyzed by confocal microscopy. To assess the number of autophagic particles per cell, cell nucleus was stained with Hoechst 33342 (blue spots). Representative images of autophagic particles were given. Stained cells with the premo-autophagy kit were either non-infected (NI cells) or infected with M. abscessus strains (Mabs wt and Δeis2MAB) or treated with chloroquine 30 μM for 48 h (+chloro) inhibiting autophagy or with HBSS solution for 2 hours (+HBSS) inducing autophagy. Autophagic particles are represented in red. Stained cells with the premo-autophagy kit were either non-infected (NI cells) or infected with M. abscessus strains (Mabs wt and Δeis2MAB) or treated with chloroquine 30 μM for 48 h (+chloro) inhibiting autophagy or with HBSS solution for 2 hours (+HBSS) inducing autophagy. Acidification of autophagosomes was determined by dividing GFP (sensitive to acidic pH) over RFP (no sensitive to acidic pH) fluorescence intensity. The number of autophagic particles per cell and acidification of autophagosomes were determined with the Fiji software. C. Phagosomal acidification was assessed as previously described [38]. Mϕ were infected at 10 (C) or 30 MOI (A and B). Histograms with error bars represent means ± SD. Differences between means were analyzed by ANOVA and the Tukey post-test allowing multiple comparisons to be performed. ns = non-significant. * p<0.05, ** p<0.01, *** p<0.001, **** p<0.0001. (TIF) [file ppat.1008069.s006.tif]
