# Supplementary material for: Mycobacterium abscessus virulence traits unraveled by transcriptomic profiling in amoeba and macrophages
Source: PLoS Pathog. 2019 Nov 8;15(11):e1008069. doi: 10.1371/journal.ppat.1008069 (PMC6839843; doi:10.1371/journal.ppat.1008069)
Supplement: S1 Table — (DOCX) [file ppat.1008069.s007.docx]

**Supp. Table 1: Differentially expressed genes identified with the *DEseq2* package.**

| **Transcriptomes** | **Down DEGs^a^** | **Up DEGs** | **Total** | **CDS^b^ cover** |
| --- | --- | --- | --- | --- |
| Mabs^c^, Ac 4 hpi | 1218 | 1355 | 2573 | 56% |
| Mabs, Ac 16 hpi | 1095 | 1122 | 2217 | 49% |
| Mabs, Mφ 16 hpi | 1646 | 1671 | 3317 | 73% |
| Mchel^d^, Ac 16 hpi | 1186 | 1949 | 3835 | 80% |

**^a^**DEG: Differentially expressed genes.

^b^CDS: Coding DNA Sequence.

^c^Mabs: *M. abscessus* subspecies *abscessus* CIP 104536T.

^d^Mchel: *M.chelonae* CCUG 47445.
