# Supplementary material for: Mycobacterium abscessus virulence traits unraveled by transcriptomic profiling in amoeba and macrophages
Source: PLoS Pathog. 2019 Nov 8;15(11):e1008069. doi: 10.1371/journal.ppat.1008069 (PMC6839843; doi:10.1371/journal.ppat.1008069)
Supplement: S2 Table — (DOCX) [file ppat.1008069.s008.docx]

**Supp. Table 2: List of *M. abscessus* genes highly induced in Ac only.**

| **Mma gene^a^** | | **Encoded protein** | **IPS^b^ analysis** | **Mabs^c^ BBH** | **MTB^d^ BBH** | **MAV^e^**  **BBH^f^** | **FC^g^ Ac, 4 hpi** | **FC Ac, 16 hpi** |
| --- | --- | --- | --- | --- | --- | --- | --- | --- |
|  | ***Highly induced genes 4 hpi*** | | | | | |  |  |
| *MYCMA_RS01100* | | Hypothetical protein | No IPS | *MAB_4664** |  |  | 5.71  (7.18E-18) | NC^h^ |
| *MYCMA_RS08980* | | Glyoxalase |  | *MAB_3056c* |  |  | 5.32  (1.79E-13) | 1.81  (1.44E-02) |
| *MYCMA_RS10865* | | Hypothetical protein | Magnesium transporter MgtE (006669) | *MAB_2717c* |  | *MAV_2122* | 5.26  (6.90E-29) | 1.44  (2.61E-03) |
| *MYCMA_RS14505* | | MULTISPECIES: 3-methyl-2-oxobutanoate hydroxymethyltransferase |  | *MAB_1916c* | *panB* | *panB* | 5.22  (2.61E-03) | 1.89  (5.08E-03) |
| *MYCMA_RS17210* | | Copper oxidase |  | *MAB_1271c* |  |  | 4.99  (5.08E-03) | 2.71  (6.51E-06) |
| *MYCMA_RS10860* | | Hypothetical protein | No IPS | - |  |  | 4.99  (1,78E-20) | 1.26  (2.05E-02) |
| *MYCMA_RS11360* | | Monooxygenase |  | *MAB_2607* |  |  | 4.93  (1.78E-20) | NC |
| *MYCMA_RS10755* | | Hypothetical protein | No IPS | *MAB_2738c* |  |  | 4.84  (1.41E-09) | 2.22  (2.00E-05) |
| *MYCMA_RS17970* | | Hypothetical protein | No IPS | *MAB_1117c* |  |  | 4.49  (5.71E-21) | 2.33  (1.13E-06) |
| *MYCMA_RS07655* | | MULTISPECIES: alkylhydroperoxidase |  | *MAB_3311c* |  |  | 4.48  (6.62E-14) | 3.25  (2.66E-07) |
| *MYCMA_RS02005* | | Short-chain dehydrogenase |  | *MAB_4478c* |  | *MAV_1797* | 4.45  (1.76E-10) | 1.84  (1.43E-02) |
| *MYCMA_RS06830* | | Nitroreductase |  | *MAB_3461c* |  |  | 4.38  (5.94E-13) | 2.11  (5.48E-04) |
| *MYCMA_RS20755* | | Membrane protein | Transport accessory protein MmpS (008693) | *MAB_0477* |  |  | 4.31  (1.60E-25) | 2.77  (1.41E-08) |
| *MYCMA_RS09135* | | Hypothetical protein | Virulence factor BrkB (017039) | *MAB_3025* | *Rv2707* | *MAV_3600* | 4.29  (1.43E-22)  ) | 1.90  (1.63E-05) |
| *MYCMA_RS11655* | | Hypothetical protein | No IPS | - |  |  | 4.23  (4.28E-11) | 1.87  (3.68E-03) |
| *MYCMA_RS08985* | | Pyridoxamine 5'-phosphate oxidase |  | *MAB_3055c* |  |  | 4.16  (1.08E-10) | NC |
| *MYCMA_RS11985* | | Sulfite reductase subunit alpha |  | *MAB_2492* |  |  | 4.13  (2.43E-15) | 2.55  (1.21E-06) |
| *MYCMA_RS12840* | | Hypothetical protein | No IPS | *MAB_2313* |  |  | 4.07  (4.69E-53) | 3.46  (5.37E-34) |
| *MYCMA_RS03450* | | Acyl-CoA dehydrogenase |  | *MAB_4158* | *fadE26* | *MAV_0652* | 4.05  (2.21E-14) | 2.31  (1.63E-05) |
|  | ***Highly induced genes 4 & 16 hpi*** | | | | | |  |  |
| *MYCMA_RS18820* | | Sugar translocase |  | - |  |  | 6.52  (1.45E-37) | 6.22  (1,42E-29) |
| *MYCMA_RS11145* | | MULTISPECIES: MmpS family protein |  | *MAB_2649** |  |  | 4.73  (1.09E-04) | 6.82  (1.42E-29) |
| *MYCMA_RS19885* | | Short-chain dehydrogenase |  | *MAB_0646c* | *Rv0068* | *MAV_4710* | 5.67  (1.09E-04) | 5.27  (7.07E-07) |
| *MYCMA_RS01105* | | Hypothetical protein | No IPS | *MAB_4663* |  |  | 6.16  (1.15E-27) | 4.18  (1.94E-07) |
| *MYCMA_RS00455* | | Hypothetical protein | Twin-arginine translocation pathway, signal sequence (006311) | *MAB_4791c** |  |  | 5.17  (4.08E-53) | 4.96  (3.03E-40) |
| *MYCMA_RS21900* | | TQXA domain-containing protein |  | *MAB_0219* |  | *MAV_2053* | 5.33  (4.62E-69) | 4.73  (1.57E-51) |
| *MYCMA_RS07540* | | MULTISPECIES: ATP-binding protein |  | *MAB_3325c* |  |  | 5.03  (3.68E-33) | 4.75  (9.87E-30) |
|  | ***Highly induced genes 16 hpi*** | | | | | |  |  |
| *MYCMA_RS13965* | | (Fe-S)-cluster assembly protein |  | *MAB_2020c* |  |  | 2.77  (1.34E-07) | 4.93  (5.09E-20) |
| *MYCMA_RS17325* | | Hypothetical protein | No IPS | *MAB_1244c** |  |  | 3.11  (9.45E-07) | 5.03  (1.90E-14) |
| *MYCMA_RS06800* | | MerR family transcriptional regulator |  | - |  |  | 2.71  (2.22E-07) | 4.00  (2.82E-14) |
| *MYCMA_RS15995* | | TetR family transcriptional regulator |  | *MAB_1518* |  | *MAV_4046* | 1.52  (5.88E-03) | 4.01  (4.07E-13) |
| *MYCMA_RS18825* | | MarR family transcriptional regulator |  | *MAB_0925c* |  |  | 3.20  (5.0E-06) | 5.04  (2.58E-12) |
| *MYCMA_RS17330* | | Hypothetical protein | ABC transporter, FecCD/TroCD-like | *MAB_1243c** |  |  | 2.86  (6,46E-05) | 4.97  (5,36E-12) |
| *MYCMA_RS17315* | | Hypothetical protein | Alkaline shock protein Asp23 (05531) | *MAB_1247c** |  |  | 3.43  (9.97E-06) | 5.10  (5.89E-11) |
| *MYCMA_RS02820* | | MULTISPECIES: molecular chaperone DnaK |  | *MAB_4273c* | *dnaK* | *dnaK* | 2.63  (4.26E-04) | 4.87  (2.58E-12) |
| *MYCMA_RS12505* | | Activator of HSP90 ATPase |  | *MAB_2387* |  |  | 3.12  (6.33E-11) | 4.99  (7.00E-11) |
| *MYCMA_RS20640* | | Carboxymuconolactone decarboxylase |  | - |  |  | NC | 7.63  (7.00E-11) |
| *MYCMA_RS10645* | | YrbE family protein |  | - |  |  | NC | 4.71  (5.73E-10) |
| *MYCMA_RS16000* | | GlcNAc transferase |  | *MAB_1517c** |  | *MAV_4048* | NC | 4.31  (7.21E-10) |
| *MYCMA_RS17320* | | Hypothetical protein | No IPS | *MAB_1246c** |  |  | 2.07  (3.80E-03) | 4.27  (3.56E-09) |
| *MYCMA_RS09170* | | GntR family transcriptional regulator |  | *MAB_3018* | *Rv0586* | *MAV_4554* | NC | 5.71  (2.99E-08) |
| *MYCMA_RS10650* | | Membrane protein | No IPS | - |  |  | NC | 4.17  (5.77E-08) |
| *MYCMA_RS01300* | | TetR family transcriptional regulator |  | *MAB_4625* |  |  | 2.55  (2.47E-04) | 4.30  (9.24E-08) |
| *MYCMA_RS10655* | | Membrane protein | No IPS | - |  |  | NC | 4.05  (1.0E-07)  ) |
| *MYCMA_RS02345* | | MULTISPECIES: alkyl hydroperoxide reductase |  | *MAB_4408c* | *ahpC* | *MAV_2839* | 3.46  (4.34E-05) | 4.04  (1.77E-06) |
| *MYCMA_RS14940* | | Transposase |  | - |  |  | 3.22  (1.05E-04) | 4.03  (2.82E-03) |

^a^Mma: *M. abscessus* subspecies *massiliense* smooth variant (accession number NC_018150.2).

^b^IPS: InterProScan protein signature.

^c^Mabs: *M. abscessus* subspecies *abscessus* (accession number NC_010397.1).

^d^MTB: *M. tuberculosis* (accession number NC_000962.3);

^e^MAV: *Mycobacterium avium* 104 (NC_008595.1).

^f^BBH = bidirectional Best hit.

^g^FC = Fold Change.

^h^NC: no change in gene expression.

*P*-values are indicted in brackets.

*: genes selected for mutagenesis.
