# Supplementary material for: Mycobacterium abscessus virulence traits unraveled by transcriptomic profiling in amoeba and macrophages
Source: PLoS Pathog. 2019 Nov 8;15(11):e1008069. doi: 10.1371/journal.ppat.1008069 (PMC6839843; doi:10.1371/journal.ppat.1008069)
Supplement: S3 Table — (DOCX) [file ppat.1008069.s009.docx]

**Supp. Table 3: List of *M. abscessus* genes highly induced in M**φ **or Ac 16 hpi**.

| **Mma^a^ gene** | **Encoded protein** | **IPS^b^ analysis** | **Mabs^c^ gene** | **MTB^d^ BBH** | **MAV^e^ BBH^f^** | **FC^g^M**φ | **FC Ac** |
| --- | --- | --- | --- | --- | --- | --- | --- |
| *MYCMA_RS01880* | Hypothetical protein | No IPS | *MAB_4509c** | **-** | - | 5.78  (2.37E-230) | 5.11  (2.82E-03) |
| *MYCMA_RS01765* | Hypothetical protein | N-acetyltransferase Eis (016181) | *MAB_4532c** | - | - | 5.37  (0) | 2.09  (6.73E-19) |
| *MYCMA_RS13035* | MFS transporter |  | *MAB_2273* | - | - | 5.26  (8.22E-206) | 4.15  (3.33E-06) |
| *MYCMA_RS17085* | Acyltransferase |  | *MAB_1297c* | - | *MAV_4113* | 4.86  (5.34E-178) | 4.91  (1.01E-39) |
| *MYCMA_RS08590* | Transcriptional regulator |  | *MAB_3134c** | - | - | 4.54  (7.64E-104) | 1.55  (1.13E-03) |
| *MYCMA_RS08595* | Hemin transporter |  | *MAB_3133c** | - | - | 4.36  (9.29E-61) | 1.14  (1.45E-02) |
| *MYCMA_RS06600* | MULTISPECIES: transcriptional regulator |  | *MAB_3508c* | *whiB7* | *MAV_4142* | 4,24  (3.41E-109) | 4,37  (3.85E-54) |
| *MYCMA_RS02565* | Acetyltransferase |  | *MAB_4324c* | - | - | 4.18  (0) | NC^h^ |
| *MYCMA_RS05440* | Membrane protein | EamA domain (000620) | *MAB_3762* | - | - | 3.96  (4.80E-135) | 4.49  (3.85E-54) |
| *MYCMA_RS12630* | ABC transporter |  | *MAB_2355c* | - | - | 3.92  (4.13E-185) | 1.76  (6.58E-19) |
| *MYCMA_RS17930* | GNAT family acetyltransferase |  | *MAB_1125c* | - | - | 3.89  (1.34E-158) | 1.62  (1.66E-06) |
| *MYCMA_RS05315* | Hypothetical protein | No IPS | *MAB_3786c* | - | - | 3.85  (7.02E-131) | 0.90  (1.55E-02) |
| *MYCMA_RS08600* | Membrane protein | No IPS | *MAB_3132c** | *Rv2620c* | *MAV_3498* | 3.83  (1.51E-47) | NC |
| *MYCMA_RS19730* | Membrane protein | EamA domain (000620) | *MAB_0677c* | - | - | 3.83  (1.00E-130) | NC |
| *MYCMA_RS22615* | Esterase |  | *MAB_0078* | - | *MAV_3025* | 3.82  (3.64E-101) | 2.92  (4.42E-05) |
| *MYCMA_RS13590* | DEAD/DEAH box helicase |  | *MAB_2158c* | - | *MAV_2956* | 3.81  (2.98E-163) | 4.38  (6.94E-43) |
| *MYCMA_RS11580* | Universal stress protein UspA |  | - | - | - | 3.78  (1.33E-09) | NC |
| *MYCMA_RS06595* | Hypothetical protein | No IPS | *MAB_3509c* | - | - | 3.72  (9.72E-94) | 3.35  (1.07E-41) |
| *MYCMA_RS22620* | IclR family transcriptional regulator |  | *MAB_0077* | - | *MAV_3024* | 3.69  (4.34E-62) | 2.54  (6.78E-07) |
| *MYCMA_RS08355* | Membrane protein | EamA domain (000620) | *MAB_3180* | - | *MAV_0095* | 3.68  (1.04E-122) | -1.60  (4.64E-05) |
| *MYCMA_RS04790* | DNA-binding response regulator |  | *MAB_3891c* | *devR* | *MAV_4109* | 3.62  (9.64E-47) | -1.02  (1.50E-02) |
| *MYCMA_RS19020* | Hypothetical protein | FAD//NAD(P)-binding domain superfamily (I036188) | *MAB_0857* | - | - | 3.55  (2.82E-81) | 2.73  (1.93E-14) |
| *MYCMA_RS10510* | Transporter |  | *MAB_2780c* | - | - | 3.47  (2.56E-86) | 0.83  (2.40E-02) |
| *MYCMA_RS13570* | Hypothetical protein | No IPS | - | - |  | 3.45  (1.84E-08) | NC |
| *MYCMA_RS16570* | MFS transporter |  | *MAB_1409c* | *Rv1258c* | *MAV_1406* | 3.44  (2.77E-134) | 2.59  (1.81E-14) |
| *MYCMA_RS22575* | Taurine catabolism dioxygenase |  | *MAB_0086** | *Rv3406* | - | 3.41  (9.92E-09) | 5.90  (8.86E-16) |
| *MYCMA_RS19295* | Membrane protein |  | *MAB_0766* | - | - | 3.36  (4.59E-105) | NC |
| *MYCMA_RS11690* | Transcriptional regulator |  | *MAB_2562c* | *Rv0081* | *MAV_5108* | 3.32  (1.20E-67) | NC |
| *MYCMA_RS21395* | Catalase |  | *MAB_0351* | - | *katA* | 3.27  (5.74E-83) | NC |
| *MYCMA_RS00530* | SAM-dependent methyltransferase |  | - | - | - | 3.26  (5.25E-95) | NC |
| *MYCMA_RS07025* | Hypothetical protein | 2 isopropylmate synthase LeuA, allosteric (dimerization) domain superfamily (036230) | *MAB_3424c* | - | *MAV_3928* | 3.25  (1.92E-77) | 3.06  (1.79E-18) |
| *MYCMA_RS19455* | Hypothetical protein | No IPS | *MAB_0733* | - | - | 3.22  (6.19E-58) | 1.62  (1.15E-05) |
| *MYCMA_RS06265* | Alkane 1-monooxygenase |  | *MAB_3598c* | *alkB* | *MAV_4215* | 3.14  (2.75E-66) | 2.51  (3.19E-11) |
| *MYCMA_RS15610* | Guanylate cyclase | Papain-like cysteine peptidase superfamily (IPS038765) | *MAB_1591* | *Rv1118c* | *MAV_1249* | 3.14  (3.05E-85) | NC |
| *MYCMA_RS14880* | ABC transporter |  | *MAB_1846* | - | - | 3.09  (2.35E-62) | 2.68  (3.19E-11) |
| *MYCMA_RS14875* | Peptidase |  | *MAB_1847* | - | - | 3.04  (2.14E-44) | 0.87  (2.91E-28) |
| *MYCMA_RS02705* | MULTISPECIES: aminotransferase AlaT |  | *MAB_4294* | *aspC* | *MAV_4818* | 3.04  (5.89E-123) | 1.54  (1.36E-14) |
| *MYCMA_RS19450* | Hypothetical protein | Leukocidin/porin MspA superfamily (036435) | *MAB_0734** | - | - | 3.00  (6.60E-12) | 3.15  (4.08E-18) |

^a^Mma: *M. abscessus* subspecies *massiliense* smooth variant (accession number NC_018150.2).

^b^IPS: InterProScan protein signature.

^c^Mabs: *M. abscessus* subspecies *abscessus* (accession number NC_010397.1).

^d^MTB: *M. tuberculosis* (accession number NC_000962.3);

^e^MAV: *Mycobacterium avium* 104 (NC_008595.1).

^f^BBH = bidirectional Best hit.

^g^FC = Fold Change.

^h^NC: no change in gene expression.

*P*-values are indicted in brackets.

*: genes selected for mutagenesis.
